# Multiproxy analysis of stabling layers in four middle bronze age byre-houses from the site of Oppeano 4D (Verona, Italy)

**DOI:** 10.1371/journal.pone.0323724

**Published:** 2025-05-22

**Authors:** C. Nicosia, M. Dal Corso, S. D’Aquino, M. Baldan, M. Bortolini, D. Battistel, F. Polisca

**Affiliations:** 1 Department of Geosciences, University of Padova, Padua, Italy; 2 Department of Cultural Heritage, University of Salento, Lecce, Italy; 3 Department of Environmental Sciences, Informatics and Statistics, University Ca’ Foscari of Venice, Venezia, Italy; Universita degli Studi di Milano, ITALY

## Abstract

Eight juxtaposed structures dating to the Middle Bronze Age (1650–1550 cal BCE) were exposed during rescue archaeological work in Oppeano (Veneto region, NE Italy). The site, named ‘Oppeano 4D’, was waterlogged and, as such, exhibited exceptional preservation of organic materials, including wooden structural remains and plant remains in the internal accretion deposits within each structure. In the present article, the internal stratifications of four such huts have been studied by means of a multi-disciplinary protocol including soil micromorphology and micro-XRF mapping, the study of botanical macro-remains, palynology, and the analysis of faecal biomarkers through GC-MS. Geoarchaeological and geochemical methods allowed to define deposit components necessary for the interpretation of the botanical records. The analyses revealed that the Oppeano structures are in fact byre-houses, where small herbivores were penned and in which other domestic activities, such as cereal processing by means of fire and food preparation, took place. Analyses also revealed that the floodplain offered several different natural environments for pastures and the collection of hay and litter for animal herding. These included the wetlands surrounding the site, the ruderal areas close to cultivated fields and settlement, and mixed deciduous mesophilous and hygrophilous woodlands. The carpological record showed a marked contrast among charred remains, pertaining to food processing, and uncharred seeds, fruits, buds, and twigs that derive from herbivore dung and fodder/bedding material. The palynological record reflects this dichotomy between activities related to human diet and animal penning that took place inside the structures and further revealed traces of natural environments used for pastures.

## 1. Introduction

### 1.1 Stabling layers as archive for living conditions and foddering practices

**Fig 1:**
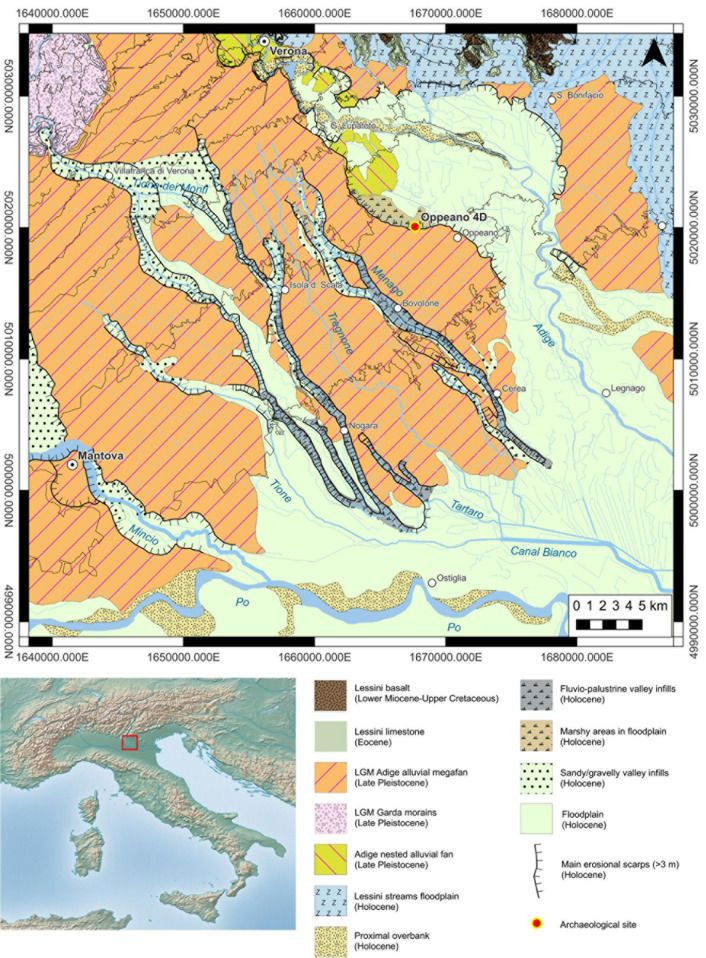
Geomorphological map showing the floodplain between the Lessini foothill and the present-day Po River. The map is based on Sorbini et al. [10], Castiglioni [119], and Carta Geologica d’Italia 1:100.000 sheets n˚ 48, 49, 62, 63 (modified from Nicosia et al. [3], fig. 1). The image of Italy in the lower left corner was obtained from Natural Earth (public domain from http://www.naturalearthdata.com).

**Fig 2:**
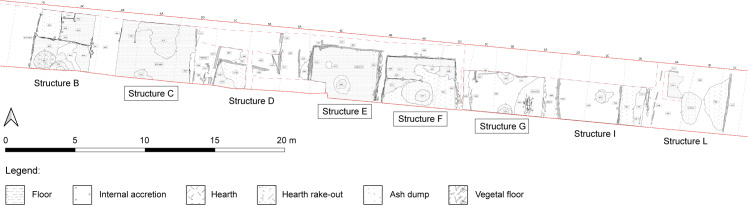
Excavation plan of the second phase of the settlement with detail on structures. Wooden elements are indicated in grey. The structures discussed in this work are indicated with a black rectangle (drawing by: M. Baldo, F. Polisca).

The case-study of Oppeano ‘site number 4D’ (Verona, Italy – hereafter, Oppeano 4D), offered an extraordinarily well-preserved waterlogged archaeological context for the investigation of living conditions and of farming and herding activities in the Middle Bronze Age (hereafter, MBA; 1650–1350/1300 cal a BCE) [1]. The latter period corresponds to a time of important demographic growth and human impact on the environment in the Po Plain of northern Italy [1,2]. At Oppeano 4D (Fig 1), eight synchronous dwelling structures or huts were exposed, featuring preserved wooden walls and finely laminated internal stratifications deriving from their daily use. Preliminary geoarchaeological analysis on the internal deposits of two such huts (structures E and F; Fig 2) allowed us to interpret them as byre-houses, due to the presence of *in situ* dung accumulations intercalated with domestic waste and hearths [3].

By applying different geoarchaeological, archaeobotanical, and geochemical methods, we aim at gaining as much information as possible from the inner stratigraphy of these structures. Specifically, the focus will be on stabling layers (also referred to as “manure layers” [4]). These are normally rather difficult to identify in the field and, as such, they are understudied archaeological contexts [5–8]. Stabling layers are organic-rich deposits mainly composed of herbivore dung, where the additional presence of other vegetal materials such as litter for bedding and/or fodder cannot be excluded. They have a high potential for yielding information in terms of living conditions within the settlement, where space was shared closely by people and their herds. Until the last century, in many rural areas in Europe, byres and dwellings have functioned as integrated domestic spaces, exhibiting less distinct differentiation than contemporary Western standards would expect [9]. Moreover, given the abundance of herbivore dung, stabling layers can offer insight on economic and environmental aspects related to plant resource management and human-environment interactions. Specifically, our research questions are:

[1] What are the micromorphological characteristics of trampled herbivore dung in waterlogged conditions?
[2] Can we differentiate between herbivore dung, bedding/fodder material, and human faecal material? What were the living conditions in the huts?
[3] Which plant components were used for animal husbandry? Can they inform us about the seasonality and the environmental impact of herding practices?

Three research questions were addressed through soil micromorphology, micro-XRF, faecal biomarkers, parasitological, palynological, and seeds/fruits analyses. Such a multidisciplinary study is carried out on four structures (Fig 2): two of them, huts C and G, are so far entirely unpublished, whereas for huts E and F only soil micromorphology and micro-XRF mapping have been already published [3].

### 1.2 The site of Oppeano 4D: geomorphology and archaeology

The site of Oppeano 4D is located in northern Italy, south-west of the city of Verona. The site lies in a low-lying basin cut into the Adige late Pleistocene alluvial fan (Fig 1) and subject to fluvio-palustrine sedimentation throughout the Holocene [10]. The repeated arrival of distal overbank and crevasse splay sediments from the Adige River to the north resulted in the deep burial of the site ca. 2 m below the present soil surface. Archaeological excavations took place in 2014–2015 due to pipeline construction, with a 70 m-long and 5 m-wide trench. Excavations led to the discovery of a MBA settlement, with different dwelling phases sealed by accumulations of peat and gyttja [11]. This work focuses on the second phase of the settlement, dating to the MBA 1–2, 1650–1430/20 cal a BCE [3]. This phase features one of the best-preserved examples of domestic structures in Mediterranean protohistory. Eight huts built on dry ground (i.e., not pile-dwellings) were in fact exposed and excavated. The waterlogged nature of the deposit allowed for the exceptional preservation of their wooden structural elements (Fig 2). Although the size of the excavation trench did not allow for the complete exposure of the structures, it was possible to establish that they were oriented E-W and had a rectangular shape with roughly similar dimensions (approximately 5 m E-W, and at least 4 m N-S). Radiocarbon dates and stratigraphic evidence indicate that the structures were built and functioned all at the same time [3]. Walls were built using the ‘post-and-wattle’ technique (cf. [12,13]), with occasional horizontally-lying large posts. This allowed for a clean definition of internal and external spaces of the structures. These acted like ‘traps’ for the anthropogenic deposits that were produced by daily activities. The internal sequences were characterised by finely laminated organic-rich sediments and ash layers deriving from hearth rake-out, with frequent replastering episodes of the internal hearths. The combined usage of the structures for animal penning and domestic activities is demonstrated by the presence of hearths, pottery fragments, burnt bones, and textile tools (i.e., spindle whorls, pins, a weaving sword) [14] associated with large quantities of *in situ*, trampled herbivore dung, as documented by preliminary micromorphological analysis on structures E and F (Fig 2) [3]. The closure of phase 2, and the end of the life of the huts, is marked by the formation of a laterally continuous layer of peat. This suggests a rather fast transition towards wetter conditions in this part of the floodplain, resulting in the sudden abandonment of the settlement[11]. No evidence for roof or wall collapse was observed, suggesting that the houses were dismantled prior to their abandonment, possibly to recover construction material in view of the successive dwelling phases at the site. The rapid burial and the onset of permanent waterlogged conditions soon after site abandonment allowed for the preservation of organic remains and hampered bioturbation. This resulted in a pristine state of preservation of stratifications and of all organic material, including uncharred plant remains.

## 2. Materials and methods

### 2.1 Sampling strategy

Stratigraphic baulks were left unexcavated across the internal area of each structure for sampling purposes. Soil micromorphology samples were collected using Kubiena boxes and gypsum bandages, following the methods of Stoops and Nicosia [15]. Bulk samples for pollen analysis and GC-MS were sub-sampled from micromorphological blocks before their impregnation to ensure a close relation with micromorphological results. A greater detail of sub-sampling was maintained for finely laminated units (ranging from 0.5–2 cm) and a slightly larger sampling pace in the case of massive layers (2–4 cm). However, subsampling always considered the boundaries between the stratigraphic units as defined in the field.

Samples for macro-remains, instead, were collected in plastic bags in the field during open area excavations. Due to the larger volume, they have a less-refined resolution than samples for biomarkers and palynology, thus often including a mixture of several fine layers of wood ash and dung-rich stabling layers (see below). The correspondence among the different samples and thin sections is explained in Table 1.

**Table 1:** List of the samples analysed in this paper for palynology, GC-MS and botanical macro-remain analyses and the corresponding micromorphological samples (Fig 3a-3b for structure C, Fig 4a-4b for structure G, SM1 for structure E, and Fig 5 and SM2 for structure F).

Structure	Unit	Brief unit description	Blocks for micromorphology	Pollen samples	Botanical macro- remains	GC- MS samples	Figures
C	488	Internal accretion composed of herbivore dung mixed with ash and charcoal	93	52, 53		43, 44	3a
	478	Internal accretion composed of ash, charcoal, and herbivore dung	93	/		42	3a
	415C	Internal accretion composed of compacted herbivore dung interfingered with ash and charcoal	52, 53	50, 51, 57, 58	415C	36, 37, 38, 39, 40, 41	3b
	382	Hearth preparation layer	/	/	382	/	/
E	619	Floor composed of organic-rich sediments	82_1	44, 45	619	/	S1
	598	Internal accretion composed of compacted herbivore dung mixed with ash and charcoal	82_3	43		/	S1
	615	Internal accretion composed of ash, charcoal, and herbivore dung	81_1 82_3	47		/	S1
	529	Internal accretion composed of ash and charcoal mixed with herbivore dung	80_1	42	529	/	S1
	578	Hearth structure	80_1, 81_3	/	578	/	S1
	308	Internal accretion composed of almost pure ash with thin laminae of herbivore dung	80_2	/	308	/	S1
F	601	Internal accretion composed of compacted herbivore dung interfingered with ash and charcoal	90, 91	72		48, 49, 50	5
	587	Internal accretion composed of ash, charcoal, and herbivore dung	91	70	587	/	5
	456	Internal accretion composed of compacted herbivore dung mixed with ash and charcoal	75, 76	48	456A-B	/	S2
	594	Localised ash dump	/	/	594	/	/
	606	Remnant of a mineral floor	/	/	606	/	/
	608	Remnant of a mineral floor	/	/	608	/	/
	632	Floor composed of organic-rich sediments, with localised enrichment of charcoal and ash	/	/	632	/	/
	563	Localised dump composed of ash, charcoal, wood fragments, and pottery fragments	/	/	563	/	/
	555	Localised dump composed of ash, charcoal, wood fragments, and pottery fragments	/	/	555	/	/
	585	Localised dump of vegetal material and pottery fragments	/	/	585	/	/
G	646 = 658	Internal accretion composed of compacted herbivore dung	103	32	658	32	4a
	654	Internal accretion composed of bedding material mixed with herbivore dung	101	29	654	27, 28, 29	4b
	635	Internal accretion composed of bedding material mixed with herbivore dung	100	28	635	25, 26	4b
	610	Internal accretion composed of bedding material mixed with herbivore dung	99	27		22, 23, 24	4b
	567	Internal accretion composed of bedding material mixed with herbivore dung, ash and charcoal	99	25		21	4b
	679	Internal accretion consisting of ash and charcoal	/		679		/

### 2.2 Micromorphology and micro-XRF

Samples were air dried (i.e., no acetone replacement) and manufactured following the methods of Murphy [16]. The micromorphological description followed the terminology proposed by Stoops [17]. Thin section analysis was carried out in plane polarised light (PPL), cross-polarised light (XPL), and observing the autofluorescence when excited with blue light (BLF). According to the preliminary micromorphological data published in Nicosia et al. [3], Soil/sediment Microfabric Types (hereafter, SMT) were employed to describe the finely laminated deposits accumulated inside the structures. In thin section, the stratigraphic units identified in the field were further subdivided into sub-units. The use of SMTs at Oppeano is motivated by the recurrence in different sub-units, even in different structures, of sets of similar micromorphological characteristics that point to similar formation processes (cf. [18,19]). SMTs are labelled with progressive numbers, and variations within the same SMT are indicated by a small letter (e.g., SMT 1, 1a, 1b). Micro-XRF mapping was performed on uncovered thin sections at Historic England (Eastney, UK) [20].

### 2.3 Botanical macro-remains

Sediment samples for archaeobotanical analysis of seeds, fruits, other vegetative macroscopic plant parts, and charcoal were taken during excavation. Nineteen stratigraphic units referable to different contexts inside the domestic structures were selected (Table 1 in SM4) for a total of 12.08 litres of sediment. For the recovery of carpological remains (seeds, fruits, and other plant parts) water sieving was used [21,22]. The floating material was collected in a sieve with a mesh size of 300 μm while the heavy residue was collected in sieves with a mesh size of 1 mm. This allowed for the recovery of macro-remains of different size classes and preservation conditions, including the uncharred plant remains. The residue resulting from sieving was first dried and then sorted in the laboratory using a Nikon SMZ745T binocular stereo microscope with magnifications from 6.7x to 50x. The conservation status of the carpological record is quite diverse and includes charred and uncharred remains. The uncharred remains are generally well preserved due to the waterlogged conditions.

Carpological remains have been grouped into nine different categories: 1. Cereals (caryopses), 2. Cereals (chaff/straw), 3. Pulses, 4. Flax, 5. Arable weeds/*Echinochloa*, 6. Arable weeds, ruderal and nitrophilous plants, 7. Grassland plants, 8. Wetland plants, 9. Trees and Shrubs (fruits, nuts and buds). Each wild *taxon* was placed in a broad habitat group based on their potential floristic associations attested in the region [23] and on the plant communities in the flora of Veneto [24]. Legumes are very few and perhaps did not play a very important role in the diet. It was decided to keep separated the *taxon* of barnyard grass (*Echinochloa crus-galli*), which is very abundant in the carpological record and whose interpretation is debated. Although the plant is a wild weed, it could also be used as cereal of economic interest for nutrition. Each remain was counted as one, even when only a fragment was found.

### 2.4 Palynology

Palynological analysis focused on the stabling layers within the studied structures (19 samples) (Table 1, Fig. 34–5). An amount of 1–4 g of material per each sample was prepared following the procedure described in Dal Corso [25], except for the use of ultrasonic device for clay removal. Percentage values have been calculated on a pollen sum of >300 grains of Spermatophytes including wetland plants. For fern spores, NPPs and other excluded *taxa*, percentages have been obtained on the sum of total Spermatophytes plus each excluded *taxon* itself. Pollen conglomerates of the same pollen type or pollen clumps [26], derived by the presence of an anther, have been counted as a single unit since the precise number of single pollen grains was mostly uncountable. For pollen identification, standard keys of European pollen flora [27,28] and pollen atlases [29–31] have been used, in addition to the comparison with reference material of the Palynology Laboratory of Kiel University. Cerealia-type pollen has been distinguished from wild Poaceae pollen based on size > 37 µm, with *porus* and *annulus* > 11 µm [32,33]. When possible, a distinction in *Triticum*-type, *Hordeum*-type and *Avena*-type was based on *exine* ornamentation [27]. The identification of NPPs relies on literature (e.g., [34–36]) and online atlases of the University of Göttingen (https://non-pollen-palynomorphs.uni-goettingen.de/), of Kiel University (https://www.wikis.uni-kiel.de/non_pollen_palynomorphs/doku.php/home) and of a parasitology website (https://www.microbiologiaitalia.it/parassitologia). Latin plant names follow the regional flora [24], which also provided flowering period, ecological, and ethnographic information.

### 2.5 Biomarker analysis

**Fig 3:**
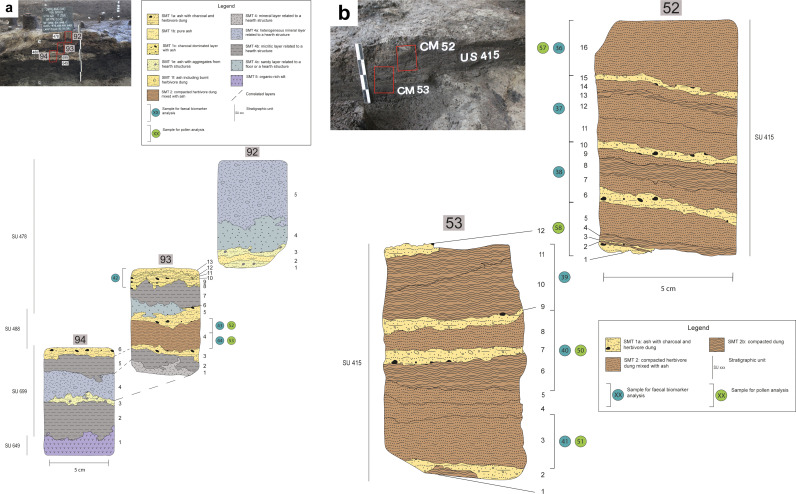
Structure C. Sampling location of samples 92-94 (a) and 52-53 (b). (a) Top left: position of samples 92, 93, and 94. Lower left and right: interpretation of the thin sections, with a SMT assigned to each sub-unit and the location of subsamples for pollen and faecal biomarker analysis. (b) Top left: position of samples 52 and 53. Lower left and right: interpretation of the thin sections, with a SMT assigned to each sub-unit and the location of subsamples for pollen and faecal biomarker analysis.

**Fig 4:**
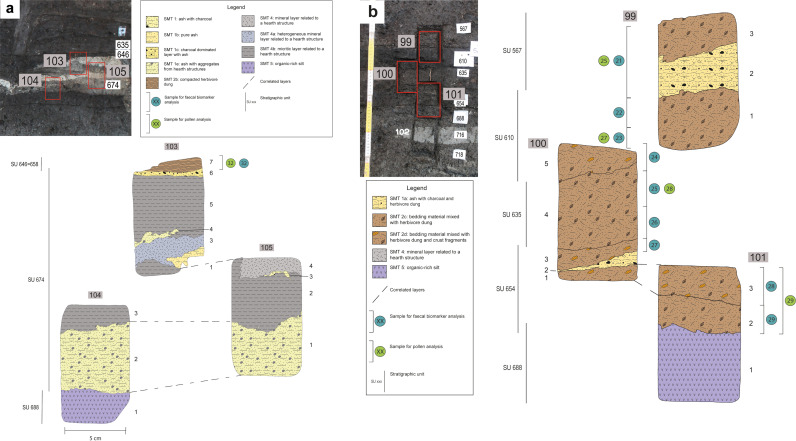
Structure G. Sampling location of samples 103-105 **(a)** and 99-101 **(b)**. (**a)** Top left: position of samples 103, 104, 105. Below: interpretation of the thin sections, with a SMT assigned to each sub-unit and the location of subsamples for pollen and faecal biomarker analysis. (**b)** Top left: position of samples 99, 100 and 101. Right hand side: interpretation of the thin sections, with a SMT assigned to each sub-unit and the location of subsamples for pollen and faecal biomarker analysis.

**Fig 5:**
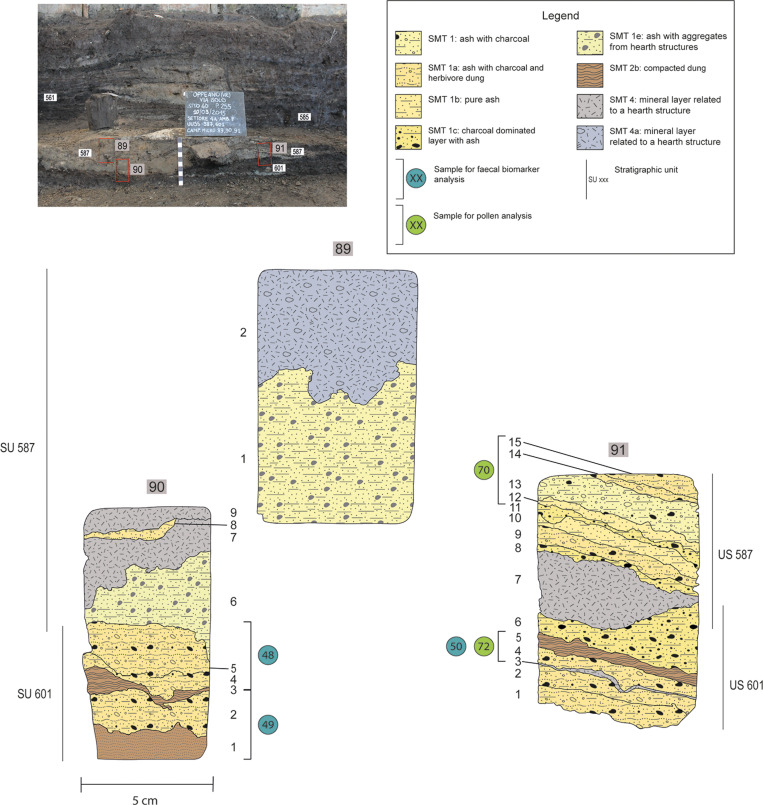
Structure F. Sampling location of samples 89-91 in the lower portion of the internal stratification of structure F. Upper left: field picture showing the position of samples 89, 90, and 91. Below: interpretation of the thin sections, with a SMT assigned to each sub-unit and the location of subsamples for pollen and faecal biomarker analysis.


Investigation of faecal steroids was carried out through GC-MS (Gas Chromatography-Mass Spectrometry) on 24 samples of sediments collected from three of the studied structures (C, G and F, see Table 1). The correspondence between the stratigraphy and the sampling spots is shown in Fig 3–5. Moreover, a set of reference samples from crops (seeds, ears, stems, and inflorescences) and leaves of trees representative of the Bronze Age was analysed. The analytical procedure used for both archaeological sediments and plant material followed Battistel et al. [37] for faecal biomarkers analyses, with some modifications detailed in Bortolini et al. [38]. The analytical procedure of sample preparation and the instrumental settings are reported in Table 1 in SM6, as well as the list of the investigated steroids with the related m/z ions used for the analysis. Four diagnostic indexes (R1-R4) based on faecal steroid ratios were used to improve the interpretation of the results. These indexes are defined as follows: [39] [40] [41] [38]

$$R1=\frac{coprostanol+epi-coprostanol}{coprostanol+epi-coprostanol+5\alpha -cholestanol}$$

$$R2=\frac{5\beta -stigmastanol+epi-5\beta -stigmastanol}{5\beta -stigmastanol+epi-5\beta -stigmastanol+5\alpha -cholestanol}$$

$$R3=\frac{coprostanol}{coprostanol+5\beta -stigmastanol}$$

$$R4=\frac{coprostanol+epi-coprostanol}{coprostanol+epi-coprostanol+5\beta -stigmastanol+epi-5\beta -stigmastanol}$$

## 3. Results

### 3.1 Micromorphology and micro-XRF

The internal stratifications of all four analysed structures yielded similar micromorphological characteristics. In general, the finely laminated units that compose these internal stratifications consist predominantly of fresh (i.e., unburnt) herbivore dung (SMT 2 and sub-types) and of wood ash (SMT 1 and sub-types). These SMTs are discussed in detail in this paper, as the latter focuses on stabling layers. Other SMTs which pertain to tar levels, hearth preparations, and backfill units (SMT 3, 4, and 5 respectively) are only summarised in Table 2.

**Table 2:** Main characteristics and interpretation of each SMT and subtype.

SMT	Main characteristics			Interpretation
**Microstructure**	**Diagnostic inclusions**	**Orientation coarse components**	
**1**	Massive	Wood ash Charcoal Vitrified phytoliths Chaff Bone fragments	Parallel and horizontal	Hearth rake-out and trampling. Wood as main combustible
**1a**	Massive	Same as 1 Herbivore dung Mineral trample	Parallel and horizontal	Same as 1 with presence of herbivores close to the structure
**1b**	Massive	Wood ash Fine charcoal Articulated phytoliths	Parallel and horizontal	Same as 1, but in more oxidising burning conditions
**1c**	Massive	Charcoal Wood ash	Parallel and horizontal	Same as 1, but in more reducing burning conditions
**1d**	Massive	Fecal spherulites Bone fragments	/	Same as 1a
**1e**	Massive	Same as 1b Aggregates of SMT 4	Parallel and horizontal	Same as 1, but also destruction and reconstruction of hearths
**1f**	Massive	Same as 1 Burnt herbivore dung	Parallel and horizontal	Same as 1, but use of herbivore dung as combustible
**2**	Microlaminated undulating	Herbivore dung Wood ash Charcoal Bone fragments Mineral trample	Parallel and horizontal	*In situ* stabling activities. Trampled
**2a**	Subangular blocky	Herbivore dung Complete ovicaprid excrement pellets	Random	*In situ* stabling activities. Non trampled
**2b**	Microlaminated undulating	Herbivore dung	Parallel and horizontal	*In situ* stabling activities. Trampled
**2c**	Microlaminated to channel	Vegetal tissues/organ Articulated phytoliths Herbivore dung Wood fragments charcoal	Moderate to strong horizontal orientation	*In situ* stabling activities (herbivore dung + bedding/fodder material). Trampled
**2d**	Microlaminated to channel	Same as 2c Stable crust fragments	Moderate to strong horizontal orientation	*In situ* stabling activities (herbivore dung + bedding/fodder material). Trampled
**3**	Massive	Wood tar Charcoal Plant residues	Random	Wood tar production
**4**	Massive	Quartz Mica Micritic aggregates	Mica shows preferential orientations	Hearth preparation
**4a**	Massive	Same as 4 Dismantled hearth preparations Pottery fragments Overbank calcareous clay-silt aggregates	Same as 4	Heterogeneous hearth preparation
**4b**	Vughy to massive	Micrite Quartz	/	Combustion surface of hearth preparation
**4c**	Single grain	Well sorted limestone fragments Quartz Chert Plagioclase	/	Floor or hearth preparation
**5**	Massive	Vegetal tissues and fragments Quartz Mica Charcoal	Random	Organic-rich floor or ground raising layer

#### Trampled dung deposits (SMT 2 and sub-types).

**Fig 6:**
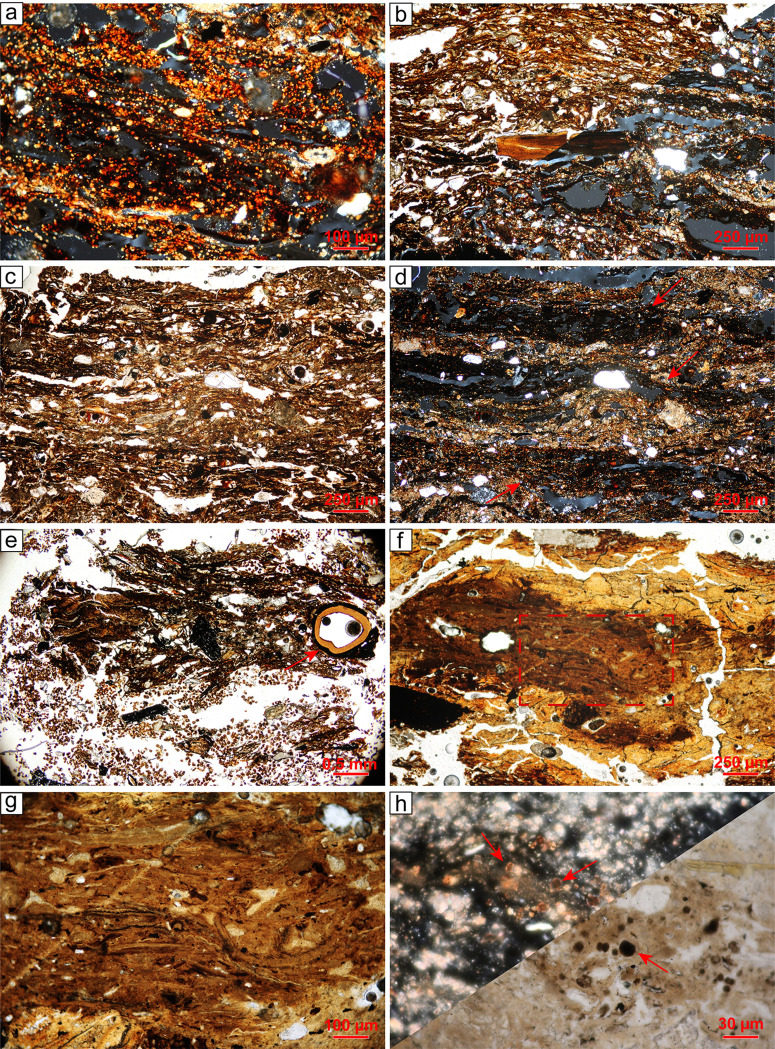
(a) structure C, thin section 52, SU 415 C, sub-unit 8: trampled, fresh (i.e., unburnt) herbivore dung. Notice the presence of numerous faecal spherulites, XPL; (b) structure C, thin section 52, SU 415 C, sub-unit 12: snapped burnt bone fragment embedded in a groundmass consisting of herbivore dung intercalated with wood ash, PPL (upper left) and XPL (lower right); (c) structure C, thin section 52, SU 415 C, sub-unit 7: Herbivore dung interfingered with and wood ash. Note the pronounced horizontal orientation of coarse components, PPL; (d) same as ‘c’ but XPL. The distinction between dung and ash is evident due to the undifferentiated b-fabric of organic matter and the crystallitic one of wood ash; (e) structure G, thin section 101, SU 654, sub-unit 2: in the upper part, vegetal material and a seed (arrow) preserving a horizontal orientation; in the lower part, bioturbated area with excrements of mesofauna without any evidence of aging, PPL; (f) structure G, thin section 100, SU 654, sub-unit 3: stable crust with a phosphate micromass that includes articulated and sub-horizontally oriented phytoliths. The rectangle indicates the location of ‘g’, PPL; (g) detail of ‘f’, showing the presence of articulated phytoliths in a phosphate micromass, PPL; (h) structure C, thin section 93, SU 478, sub-unit 10: aggregate of burnt herbivore dung embedded in an ash groundmass. The red arrows indicate examples of blackened faecal spherulites, PPL (lower right) and XPL (upper left).

Herbivore dung is composed of articulated phytoliths and elongated brownish vegetal tissues associated with faecal spherulites in various amounts, sporadic diatoms, and *Chrysophyceae* cysts (Fig 6a). The abundance of faecal spherulites -calcite crystals that form in the gut of herbivores [42]) - suggests a faecal input derived from ovicaprids and/or bovids. These domestic animals are in fact the ones that produce the most faecal spherulites, while horses usually do not produce them [43]. No other domestic herbivore has been identified in the zooarchaeological record at Oppeano 4D [44]. The presence of ovicaprids is further confirmed by sporadic faecal pellets, entire or fragmented, characterised by a convolute internal morphology and a thicker outer rim [45](cf. [45]). In general, herbivore dung units and sub-units are compact and have a microlaminated undulating microstructure [46,47]. This derives from *in situ* trampling that leads to compression and horizontal arrangement of vegetal remains [48].

**Fig 7:**
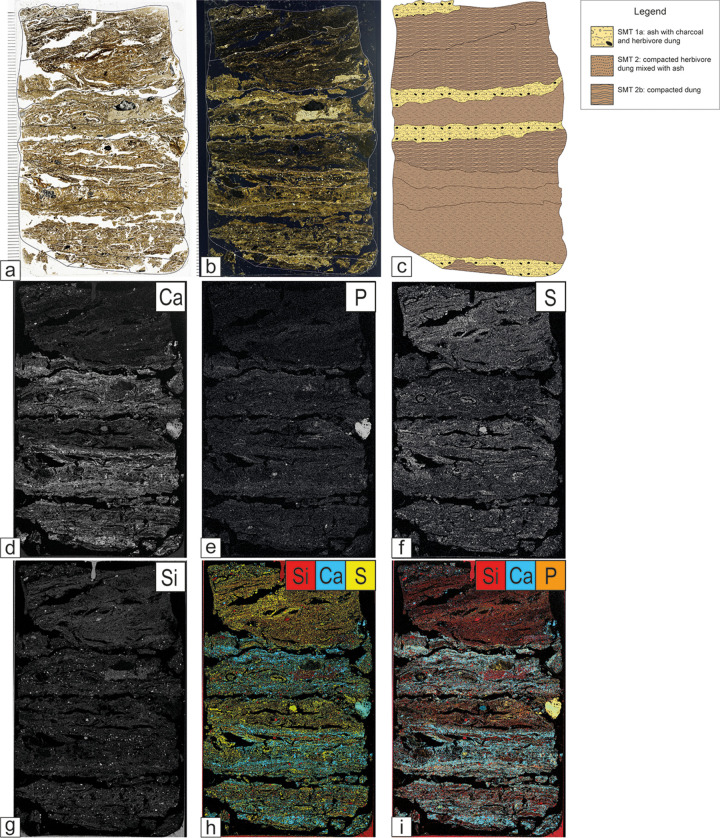
Structure C, thin section 53. Scanned thin section, micromorphological interpretation and micro-XRF maps. (a) PPL scan; (b) XPL scan; (c) interpretation of the thin section; (d-i) micro-XRF maps showing the abundance of specific elements.

Trampled dung deposits can be composed entirely of herbivore dung (SMT 2b) or have it as a predominant component (SMT 2). In the latter case, dung is intermixed with combustion by-products (i.e., wood ash, charcoal, molten phytoliths) as well as waste derived from food preparation such as burnt bones and fishbones, and shells (Fig 6b–d). Geogenic sediments are usually rare and include quartz and mica, with minor amounts of feldspars, chert, and quartzite. In structure G (Fig 4b), herbivore dung is often mixed with leaves, twigs, seeds, bark fragments and other vegetal remains that cannot be related directly to dung due to the absence of faecal spherulites (SMT 2c, 2d; Fig 6e). Pyrite microcrystals and framboïds typically occur in association with organic remains in SMT 2 and subtypes. The formation of pyrite likely results from the initial decay of organic matter and bacterial metabolic processes under anaerobic conditions, which lead to the reduction of sulphate and the oxidation of elemental sulphur or intermediate sulphur compounds [49]. Occasionally, phosphatic crust fragments are also documented within herbivore dung accumulations (SMT 2d). Stabling crusts typically form on stable floors due to the cementation of animal excrements and bedding or fodder as the result of phosphorus release from animal excreta [5,50]. In thin section, stabling crust fragments are yellowish/brownish in PPL, optically isotropic in XPL, and autofluorescent under blue light. They contain sporadic faecal spherulites and common articulated phytoliths that are often oriented parallel to each other (Fig 6f, g). Micro-XRF mapping confirmed that the crust fragments are composed of calcium-phosphate minerals (SM3). Excluding phosphate crust fragments, micro-XRF mapping revealed a predominance of sulphur (S) and silicon (Si) signals in stabling layers, while phosphorus (P) yielded a very low signal (Fig 7). These data corroborate what was observed in Nicosia et al. [3]. Sulphur likely derives from pyrite and organic matter, whereas Si derives mainly from phytoliths, with a minor contribution of silicates and rock fragments.

#### Ash rake-out deposits (SMT 1 and subtypes).

Micromorphology reveals the presence of ash-rich laminae derived from hearth rake-out [51]. Rake-out layers also include domestic waste, such as burnt chaff derived from cereal processing, burnt bone fragments, fishbones, and shell. Herbivore dung has been observed as a rare to common inclusion only in specific subtypes (SMT 1a, 1f), while in others it is absent (i.e., SMT 1, 1b, 1c, 1e, 1g; see Table 2). Herbivore dung is typically composed of horizontal, elongated vegetal tissues and articulated phytoliths associated with faecal spherulites, as observed in SMT 2 and sub-types (see above). Rarely, it occurs as reworked aggregates likely transported by trampling or derived from cleaning activities.

Burnt herbivore dung is very rare and limited to specific sub-units (i.e., SMT 1f). It appears as a yellowish, optically isotropic micromass with blackened spherulites (Fig 6h). Experimental research demonstrated that blackened spherulites form in reducing conditions between 500 and 700°C [52].

### 3.2 Botanical macro-remains

**Fig 8:**
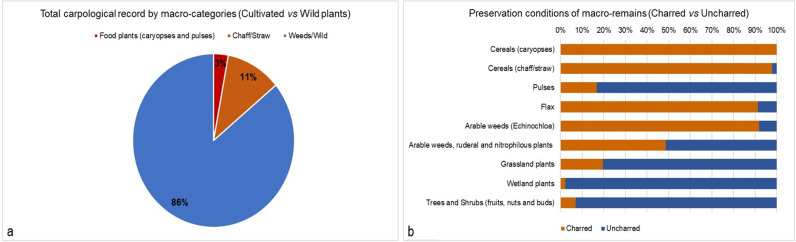
(a) Cultivated *vs* wild plants in the total carpological record; (b) Different preservation conditions of the carpological record by plant groups (charred versus uncharred botanical macro-remains).

**Fig 9:**
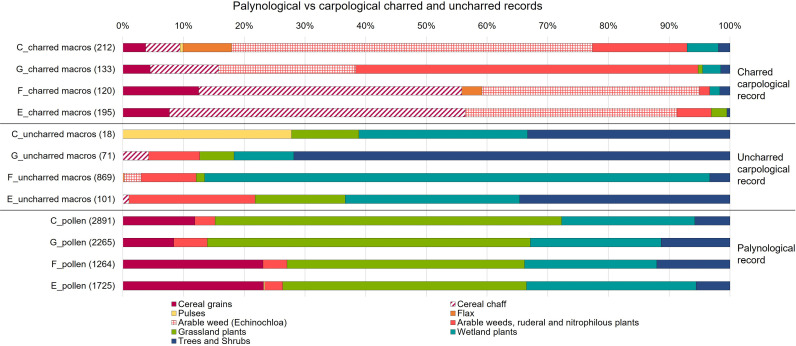
Comparison between the palynological record, the charred, and the uncharred carpological records from structures C, G, F, and E. Numbers between brackets indicate the sum of remains on which percentages were calculated. For the components of the ecological or functional botanical groups, see respectively SM4 for the carpological record and SM5 for pollen. For pollen, in the category of Grassland plants all taxa of the subgroups indicated with * in Fig 10a are included.

A total of 1709 carpological remains from 19 stratigraphic units of structures C, G, E, F were recovered and analysed (Fig 8a and SM4). The assemblage is quite diverse in terms of preservation conditions (Fig 8b) and floristic diversity: it consists of 60 plant taxa grouped into nine categories (SM 4). The carpological results found in each structure are shown in Fig 9, where charred and uncharred remains are displayed separately.

Overall, the best represented category in quantitative terms is that of wetland plants (n. 781). This data is conditioned by the numerous unburnt sedge seeds in one level (SU 606) of structure F. However, the presence of plants typical of humid environments and of river banks is constant in all the structures and includes different species of sedges (*Carex* cf. *acuta*, *acutiformis* or *Carex distans*, and *C*. *hirta*), gypsywort (*Lycopus europaeus*), marsh woundwort (*Stachys palustris*) buttercups and knotweed (e.g., *Ranunculus sardous*, *Persicaria lapathifolia*). Almost all seeds in this category are uncharred.

Another well-attested category in all the structures is that of arable weeds, ruderal, and nitrophilous plants (n. 206). This category includes those species frequently associated with crops and disturbed areas in and around the settlement. Species typical of synanthropic environments such as ruderal uncultivated lands, roadsides, areas trampled by humans and animals were also included. In percentage terms, this category is more represented in structure G. Charred and uncharred goosefoot (*Chenopodium album*) are generally more abundant and common in all structures. Also very common are carrot seeds (*Daucus carota*), black-bindweed (*Fallopia convolvulus*), cleavers (*Galium aparine*), common purslane (*Portulaca oleracea*), annual yellow woundwort (*Stachys annua*), common vervain (*Verbena officinalis*), common nettle (*Urtica dioica*). Barnyard grass caryopses (*Echinochloa crus-galli*) have been kept separate from this group. The latter is in fact a species now considered as a weed of cultivated fields but it is not excluded that in the past it could have had a food use for humans and animals. The frequent presence of both charred and uncharred barnyard grass caryopses varies in the four structures but is especially abundant in C (n. 126). Barnyard grass is a small-grained grass species of the same Panicoideae subfamily of other millets, which are very rare in this study.

Among the cereal group, caryopses (n. 44) have been distinguished from chaff/straw (n. 189). This distinction of caryopses from other cereal plant parts is fundamental for the evaluation of contextual aspects and for the reconstruction of food processing, preparation, and consumption practices. Cereals are present in every structure with notable percentage variations and almost always in charred form. In structure E, the presence of the spikelets forks is remarkable. In general, hulled cereals such as emmer (*Triticum dicoccum*), einkorn (*Triticum monococcum*, Fig 11j) and “new glume wheat” (*Triticum timopheevii sensu lato*) (Filipović *et al.* 2024) are well represented. Barley (*Hordeum vulgare/disticum*), naked wheats (*Triticum aestivum/durum*, Fig 11k), broomcorn millet (*Panicum miliaceum*) and foxtail millet (*Setaria italica*) are also attested. Broomcorn millet, and likely also foxtail millet, are crops introduced in the MBA in Northern Italy [53–56], where they are widely used for human consumption [57], but in Oppeano 4D they are particularly rare. There are numerous chaff/straw remains that have not been determined at species level (*Triticum* sp.) due to excessive fragmentation or poor preservation, that preclude good observation of the anatomical characters.

**Fig 10:**
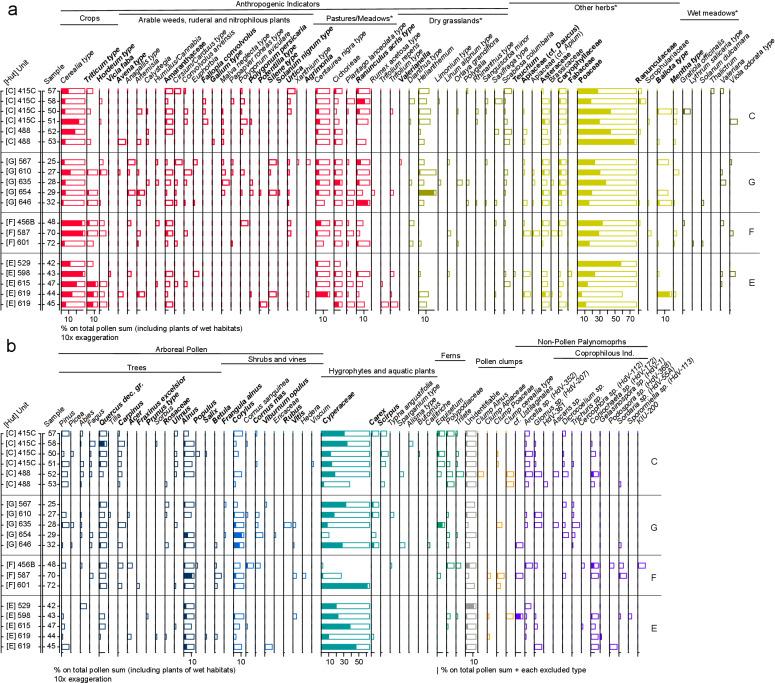
Pollen diagrams from structures C, G, F, and E of Oppeano 4D: (a) **non-arboreal pollen including terrestrial herbs (secondary anthropogenic indicators and wild plant ***taxa*); (b) arboreal pollen (including trees, shrubs, and vines) and pollen of wetland plants; (c) non-pollen palynomorphs and pollen clumps. Empty histograms display exaggeration factor 10x. Plant groups with * compose the group of “Grassland plants” in Fig 9.

**Fig 11:**
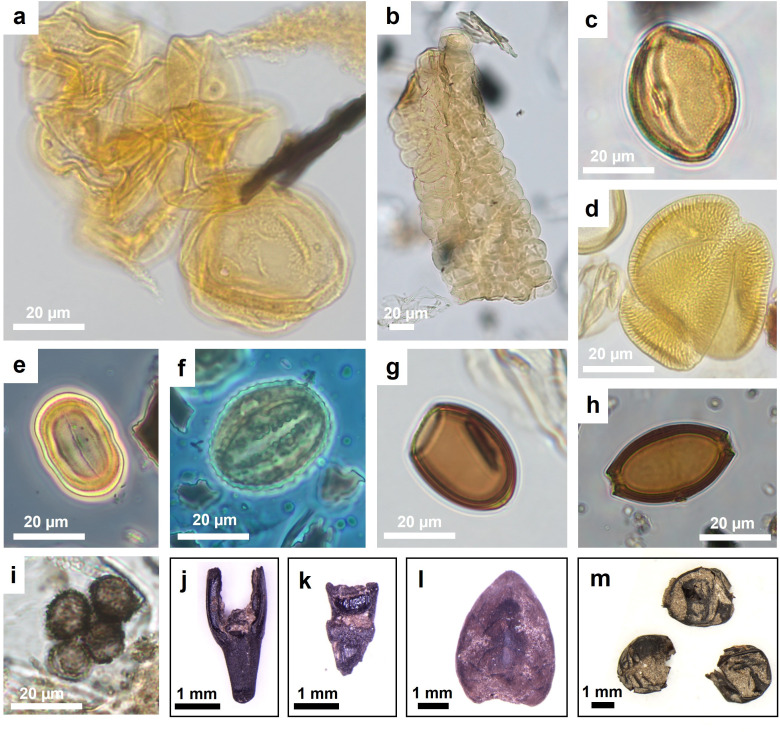
Microphotographs of microfossils from the botanical macro- and micro-remain records at Oppeano 4D: (a) pollen clump of Cerealia-type; (b) pollen clump of wild grasses (Poaceae); (c) *Helianthemum* pollen; (d) *Convolvulus arvensis* pollen; (e) *Polygonum aviculare* pollen; (f) *Centaurea nigra* type pollen; (g) *Dicrocoelium* sp. intestinal parasite egg; (h) *Trichuris* sp. intestinal parasite egg; (i) cf. Ustilaginales spores; (j) *Triticum monococcum* fork; (k) *Triticum aestivum/durum* rachis fragment; (l) bud of deciduous *Quercus* cf. *robur*; (m) *Melophagus ovinus* L. puparia.

The remains of the trees and shrubs (fruits, nuts and buds) category are 128 in total and include many edible seeds of spontaneous plants. They regularly appear in different structures and more abundantly in G. These carpological remains are mostly preserved uncharred, such as the pericarp fragments of hazelnuts (*Corylus avellana*), grape seeds (*Vitis vinifera* subsp. *sylvestris*), the endocarps of cornelian cherry (*Cornus mas*) and blackberry (*Rubus fruticosus*). The fruits of wild arboreal plants also include various parts of oak acorns (including fragments of pericarp and acorn bases). The oak (*Quercus* cf. *robur*) is also represented by the presence of fragments of uncharred terminal buds (Fig 11l)

Species from the grassland category are less present (n. 41). This group includes various species typical of meadows and grazed areas, such as Asteraceae (e.g., *Crupina* cf. *vulgaris*) or Rosaceae (*Agrimonia eupatoria, Potentilla argentea*, *Potentilla* sp.). Again, most of the remains from these wild plants were preserved in uncharred form. The latter include in Unit 619 of structure E, e.g., common agrimony (*Agrimonia eupatoria*), common crupina (*Crupina* cf. *vulgaris*), California burclover (*Medicago* cf. *polymorpha*), and cinquefoil (*Potentilla* sp.), not present in the other contexts investigated. Finally, several charred flax seeds (*Linum usitatissimum*) have also been attested, particularly in structure C.

### 3.3 Palynological record

An average of 429 pollen grains per sample were counted, excluding fern spores, other NPPs and pollen clumps (SM5). Thanks to waterlogging, the preservation of the pollen wall or exine in the stabling layers is generally good (unidentifiable pollen grains per structures on average 1.2–4.3%), apart from some folding and crumpling of large grains such as those from cereals. In addition, pollen clumps have been found that are formed by many grains of the same pollen type attesting the presence of anthers (Fig 11a, b). The average pollen concentration in the samples from stabling layers is 127,150 pollen/gram (p/g) (SM5).

In terms of floristic richness, 96 pollen types referring to Spermatophyte taxa occur in the record, which is mostly composed of non-arboreal pollen (NAP) of terrestrial herbs (62 pollen types), followed by arboreal pollen (AP) including trees, shrubs, and vines (27 pollen types) and pollen of wetland vegetation (9 pollen types). Fern spores are also attested (2 types) as well as several non-pollen palynomorphs (NPPs) that include coprophilous indicators, like some ascospores and eggs of intestinal parasites, among other microfossils (14 types). These grouping and further subgroups are displayed in the percentage diagrams in Fig 10 and in SM5. Average percentage values of ecological/functional plant groups by structure are shown in Fig 9 and discussed hereafter. Most of the pollen record originates from plants of open environments, many of which are zoophilous (i.e., insect-pollinated): the average NAP per structure ranges between 87.9% in F and 94.4% in E. Among NAP, anthropogenic pollen indicators (after [58–60]) are very abundant, between 22.6% in C and 33.3% in E. Crops, mainly constituted by cereals, have very high values in structure E (23.4%, including one grain of *Vicia* type) and in F (23.1%); slightly less in structures C (11.9%) and G (8.4%). Cereal pollen grains whenever possible have been distinguished from the general Cerealia type, attesting *Hordeum* type (barleys), *Triticum* type (wheats, especially in structure E) and *Avena* type (oat, in structure C and structure E). Barnyard grass (*Echinochloa crus-galli*), abundant in the carpological record, produces large Poaceae pollen grains [29] that here were likely included in the Cerealia type, since species identification is not possible with the main pollen keys [27,28]. Arable weeds, ruderal and nitrophilous plants of disturbed ground are well-attested (2.9–5.5%): Amaranthaceae are ubiquitous; common knotgrass (*Polygonum aviculare*, Fig 11e), hedge bindweed (*Calystegia sepium*), field bindweed (*Convolvulus arvensis* type*,* Fig 11d), buttonweed (*Malva neglecta* type) and nettle (*Urtica*) are very frequent. Plants indicators of pastures and meadows (7.1–13.9%) include the ubiquitous cichory tribe (Cichorieae), common knapweed (*Centaurea nigra* type, Fig 11f), almost ubiquitous, meadow buttercup (*Ranunculus acris* type, especially in structure C and G) and ribwort plantain (*Plantago lanceolata*, frequent in structures E and F). According to some authors, common knapweed is favoured by grazing and animal corralling [59] while other authors report its increase after mowing [60]. In all the samples of this study, plants typical of xerophytic communities on well-drained, calcareous substrate are attested (0.9–6%). These include the ubiquitous sunrose (*Helianthemum nummularium* type, Fig 11c) and small scabious (*Scabiosa columbaria*), sporadic burnet (*Sanguisorba minor*), white laceflower (*Orlaya grandiflora*), *Rhinanthus* type, *Saxifraga* type and *Polygala.* White laceflower has been observed to increase since the end of the Early Bronze Age around the Garda Lake, in accordance to the development of secondary dry grasslands after grazing pressure [61–63]. Sea-lavender (*Limonium*), which commonly grows in sandy substrates such as littoral dunes, is present in structure C and structure G. This plant reflects an extinct vegetation of sandy dunes, which were reported as an endangered environment in the region of Oppeano in the late 1950s [64]. Among the other herbs (29–44.7%), wild grasses (Poaceae) are very abundant (over 50% in some samples of structures C and E) also as clumps (Fig 11b), and in structure C, two pollen grains of wild grass with double *pori*, instead of one *porus*, have been observed. This exceptional variation of the Poaceae morphotype seems related to water stress and dry environmental conditions [65]. Species of wet grasslands and meadows (0.2–0.8%) are *Mentha* type, *Thalictrum*, *Ballota* type (including *Stachys* sp. after [27]), and *Valeriana*. Wetland plants growing around marshes and fens are mainly sedges (Cyperaceae), *Carex*, *Scirpus*, *Typha angustifolia/Sparganium, Butomus* and *Alisma* type, and are well represented in all the samples (21.6–27.9%). Fern spores (0.2–1.1%) and *Equisetum* have been found, especially in structure C. This palynological record supports the strong carpological evidence of sedges and suggests that wet habitats were exploited for herding too.

The woody component made of pollen from trees, shrubs, and vines, has very low percentage values (AP 5.6–12.1%). The arboreal pollen mostly includes trees of the mixed deciduous oaks and hornbeam association (*Quercus* dec. group, *Carpinus*, *Ulmus*, *Corylus*) typical of mesophilous woodlands of the Po Plain and the local riparian woodland dominated by alder (*Alnus*; as pollen clumps in structure C), birch (*Betula*) and willow (*Salix*). Beech (*Fagus*) pollen, likely growing on the alpine foothills [66], is rare with exception of structure C. Beech pollen, as well as the pollen of conifers (*Abies, Pinus, Picea*) that is ubiquitous with very low values (0.2–0.6%), derived from long-distance transport by wind and water in the environment around the site. Among the shrubs, hazel is frequent (*Corylus*; abundant in structure G) and rare pollen of cornelian cherry (*Cornus mas*), common dogwood (*Cornus sanguinea*), guelder rose (*Viburnum opulus*), brambles (*Rubus*) and grapevine (*Vitis*) testify open forest patches with many fruits-bearing shrubs. Two evergreen vine species, mistletoe (*Viscum*; structure C) and ivy (*Hedera*; structure F) characterise a kind of nutritious green foliage available in winter for animals [67]. Among these species, many provide edible fruits, nuts and berries, that are part of human diet and some, especially hazel inflorescences and brambles, that could have offered fodder for domestic animals too [68,69].

Among NPPs there are the ascospores produced by coprophilous fungi commonly considered indicators of dung such as *Sordaria* sp., *Sporormiella* sp., *Gelasinospora* sp., *Cercophora* sp.*, Coniochaeta* sp., *Podospora* sp. Further indicators of faecal matter are the eggs of intestinal parasites (helminths) such as *Trichuris* sp. (Fig 11h) and *Ascaris* sp., which can be found in humans and pigs [70,71], and *Dicrocoelium* (Fig 11g) that is associated with cattle and ovicaprids [70]. These two groups of coprophilous indicators, taken together, are attested in 90% of the samples (0.9%–3.9%). They are often observed in anthropogenic deposits in sites with animal and human everyday activities [60,71–73]). In some samples in structures E, F and G, fungal spores comparable to those of smut fungi, cf. Ustilaginales (Fig 11i), have been found: these fungi are plant parasitic pathogens that can affect cereals as well as sedges and other plants [36].

### 3.4 Biomarker analysis

The steroids concentrations determined in the 24 samples from structures C, G, and F are reported in Table 2 in SM6.

Despite the large variability among structures, the total amount of sterols ranged from 1.2 up to 10 µg g^-1^. These values were generally higher compared to other archaeological sites dated back to the Bronze Age such as Düren-Arnoldsweiler (Germany) (~ 1.2 µg g^-1^) [74] or in other Italian archaeological sediments in the Veneto region, where values ranging from 0.04 to 0.76 µg g^-1^ were found [38]. Although Lerch et al. [75] in the prehistoric encampment site of Ullafelsen (Austria) found a total sterol amount up to 200 µg g^-1^, it must be noted that it mainly derives from the larger contribute of β-sitosterol which is a typical plant-derived Δ^5^- sterol. For comparison, in fresh faecal samples from livestock (herbivores and pigs) as well as in humans, the total amount of steroids is about ~ 0.2–12 mg g^-1^ [38,76].

**Fig 12:**
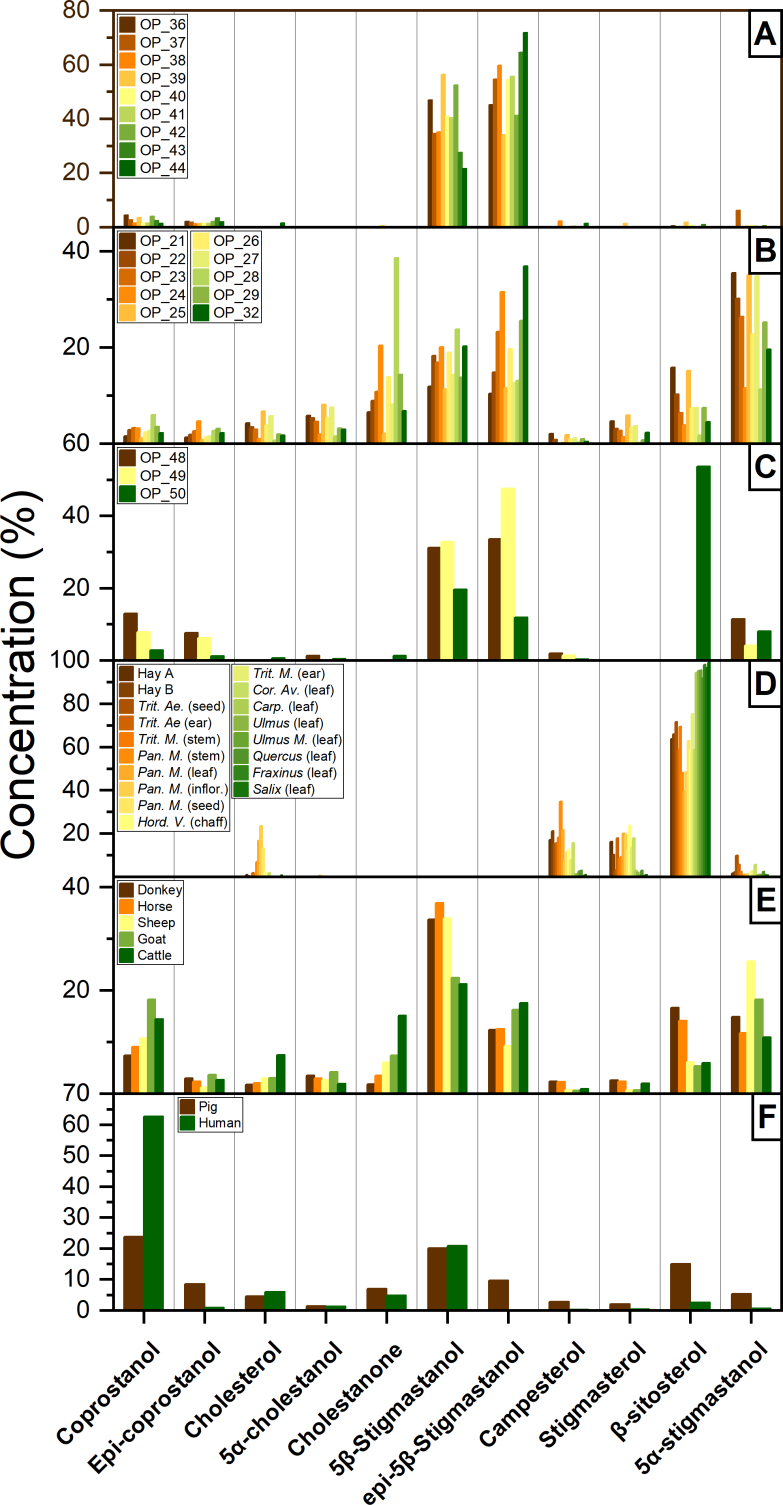
Steroid distribution from structure C **(a)**, structure G **(b)**, structure F **(c)**, plant materials **(d)** (this study), herbivores **(e)** [38], and omnivores **(f)** [74,76,120]. Concentration is reported as % on the sum of steroids.

The 5α- and 5β- congeners that derived from the reduction of Δ^5^-phytosterol (campesterol, β-sitosterol, and stigmasterol) were the most abundant steroids found in all the structures. As shown in Fig 12a–c, 5β-stigmastanol accounts for 21–56% of the total steroids in structure C, 11–24% in structure G and 20–33% in structure F. Similarly, epi-5β-stigmastanol accounts for the 34–72%, 10–37%, 12–48% of the total steroids in structure C, G and F, respectively. Moreover, 5α-stigmastanol accounts for the 0–6%, 8–35%, 4–11% of the total steroids in structure C, G and F, respectively. Phytostanols are almost absent in structure C, while they constitute the 2–23% and 1–54% in structure G and F, respectively. Finally, zoosterols (i.e., coprostanol, epi-coprostanol, 5α-cholestanol and cholesterol) are poorly represented, although coprostanol and epi-coprostanol were found in 4–20% in several samples of structure F. For comparison, the distribution of faecal steroids in several plants (this study, see Table 3 in SM6), herbivores (from [38,74,76], including ovicaprids, equines, cattle), as well as humans and pigs [74,76] are reported in Fig 12d–f.

**Fig 13:**
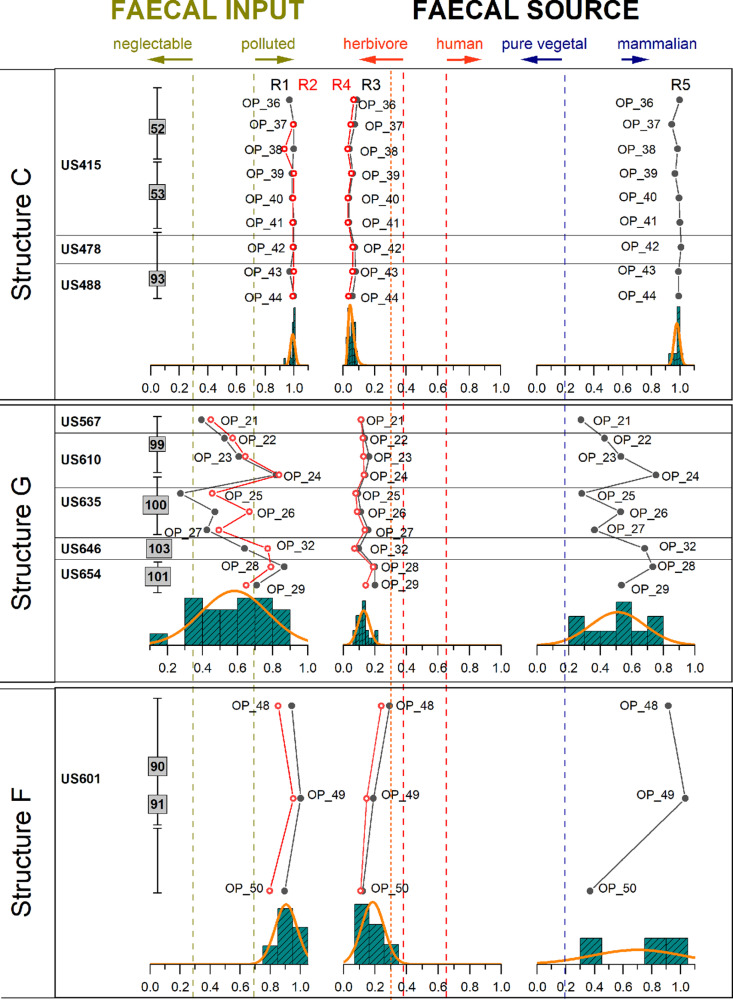
Diagnostic ratio profiles and distributions in the structures C, G and F. Dashed lines represent the limits proposed by Bortolini et al. [38], Bull et al. [40], Grimalt et al. [39], Leeming et al. [41], Prost et al. [74]. The R5 ratio and threshold are proposed in this study.

It is well established that the 5β-congeners stanols mainly derive from the reduction of Δ^5^-sterols, mediated by enteric bacteria, in the intestine of mammals [77,78]. Based on this occurrence, Grimalt et al. [39] and Bull et al. [40] proposed the use of several ratios (R1 and R2, respectively) (see Materials and Methods) as indicators of faecal input, establishing the thresholds of 0.3 and 0.7, where for values < 0.3 any significant faecal input can be excluded, while values > 0.7 are indicators of faecal pollution. In this study, R1-R2 ranges from 0.94–1 and 0.85–1 in structures C and F, respectively, while in structure G these ratios showed a higher variability (from 0.20 to 0.85, with a mean value of 0.6 ± 0.2) respectively (see Fig 13).

The predominance of 5β−phytostanols (i.e., 5β-stigmastanol and epi-5β-stigmastanol) versus 5β-zoostanols (coprostanol and epi-coprostanol) suggests the predominance of herbivore faecal input. Leeming et al. [41] and Bortolini et al. [38] proposed the use of the ratios R3 and R4, respectively to assess the predominance of an herbivore *vs* human faecal input. Similarly to R1 and R2, several thresholds have been proposed for R3 and R4, where values higher than 0.73 [41] (or > 0.65, (following [74]) can be ascribed to a predominance of human faecal input, while values lower than 0.38 [41] (or < 0.29, following [38,74]) indicate a predominance of herbivore input. In this study (see Fig 13), both R3 and R4 values were consistently lower than 0.29 in all the analysed samples. Moreover, it is worth noting that, in almost all the samples from the site of Oppeano 4D, the epi-5β-stigmastanol exceeds 5β-stigmastanol, while in fresh faecal matter the opposite is observed (see Fig 12e and Table 2 in SM6). The inverted proportions between 5β-congeners suggests that *in situ* epimerisation may have occurred due to microbiological reworking (as noted by [5,78]).

The occurrence of a high microbiological reworking, likely enhanced by the humid environment of Oppeano, might also explain the abundance of 5α-stigmastanol in conjunction with a lack of phytostanols. In fact, although 5α-stigmastanol may be produced by the metabolic activity of herbivores (mainly caprids, see Fig 12e), it generally occurrs together with the input of some phytostanols (mainly β-sitosterol). The remarkable absence of β-sitosterol together with a relatively higher abundance of 5α-stigmastanol, in conjunction with the *in situ* epimerisation of 5β-stanols, reinforces the hypothesis that strong microbiological reworking occurred in this site. In light of these considerations, the relatively higher abundance of 5α-stigmastanol may be interpreted as a consequence of the *in situ* microbial reduction of Δ^5^-phytostanols, largely present in plant materials, as reported in Fig 12d. Although *in situ* microbiological reworking strongly compromises the potential application of faecal biomarkers to distinguish between herbivore species, in the Oppeano context, a distinction between the relative proportion between dung and fodder might be established through the ratio between 5β-stigmastanol congeners *vs* phytosterols/5α-stigmastanol, defining the ratio R5 as:


$$R5=\frac{5\beta -stigmastanol+epi-5\beta -stigmastanol}{5\beta -stigmastanol+epi-5\beta -stigmastanol+campesterol+stigmastanol+\beta -sitosterol+5\alpha -stigmastanol}\;$$

We propose this ratio in this study, under the assumption that although 5α-stigmastanol is not source-specific, in the Oppeano site, the majority of 5α-stigmastanol derives from *in situ* microbial degradation. Therefore, we considered that R5 ratios in pure herbivore faecal matter is typically higher than 0.40 (data from [38]), while in plant material R5 is close to zero, as plants do not produce significant amount of 5β-congeners (see Table 3 in SM6). Therefore, with major assumptions, R5 gives a broad indication of the dung *vs* fodder predominance input in the structures that are assumed to be a closed system. Here, R5 ranges from 0.94–1, 0.28–0.75, and 0.34–0.94 in structures C, G, and F, respectively, with values generally close to the corresponding pure herbivores (0.35–0.85), but significantly higher than plant material (i.e.,* *< 0.0002).

## 4. Discussion

### 4.1 Formation of the records of botanical macro- and micro-remains

The carpological record derived from large bulk sediment samples that encompassed several micro-stratified deposits made of ash rake-outs and dung-rich/bedding accumulations, hence it represents a mixture of materials with different origins. Charred plant remains formed after deliberate or accidental carbonisation of material by humans in hearths, in relation to cereal processing and food preparation/consumption. Soil micromorphology proved that the burning of dung was not a regular practice at the site, where wood was the main fuel source (see the anthracological study in [79]). Differently, uncharred plant macro-remains, mostly waterlogged, and few mineralised seeds/fruits/other vegetative parts, were likely transported inside the structures through herbivore dung and litter/fodder, although we cannot exclude other human activities (especially for edible seeds/fruits) and accidental transport attached, e.g., to fur or hooves. In fact, carpological remains from the categories of cultivated crops and crop by-products are mostly charred. Instead, waterlogged carpological remains, likely originated from dung and litter/fodder, come from the categories of wild plants from wetlands and grasslands (i.e., pastures and meadows), as well as from the groups of arable weeds, ruderal and nitrophilous plants, and from trees and shrubs.

The palynological record in the stabling layers does not originate from the pollen rain as in natural archives in open environments, where wind-pollinated plants, e.g., trees, are over-represented. Pollen derives, instead, from plant uses in activities carried out in the structures, such as the penning of domesticated animals and food preparation. The main sources of pollen and spores are therefore plants related to pastures, animal fodder and litter, entered as such or ingested outside and deposited within dung. Overall, such pollen spectra can therefore help to reconstruct plant resources and the kinds of vegetation used for animal herding. Nevertheless, we cannot exclude some transport of pollen attached to animal fur and pollen related to plants used for human consumption or other purposes. In addition, the cereals found in Oppeano 4D have autogamous pollination and pollen was released during shattering of the ears while processing. Other two features characterising the palynological record from the stabling layers are the presence of clumps of pollen grains of the same type and the high pollen concentration. Pollen clumps are present also in ovicaprine droppings at the pile-dwelling of Lavagnone, Brescia (Appendix 5a in [80]) and likely correspond to the ingestion of inflorescences by animals, often found in dung (see also, e.g., [81–83]). In structure E, particularly high pollen concentration (average of 244,797 p/g) finds comparison with that in ovicaprine dung from the MBA site of Castellaro del Vhò, Cremona (270,000 p/g) [84].

### 4.2 Integration of different proxies by structure

#### Structure C.

Most of the stratigraphic sequence consists of an alternating sequence of *in situ* trampled herbivore dung and ash rake-out layers from hearths. These contain burnt animal bones and charred seeds of arable weeds and edible plants. The macrobotanical record is composed of abundant charred barnyard grass caryopses and goosefoot seeds, accompanied by some of the very rare charred linseed and pea remains attested at the site. This record indicates that a broad spectrum of plant-based food resources was known at the site, including wild ones and cultivars, among which also oil (and fiber) plants as flax and legumes.

In SMTs that in thin section appeared to be dung-rich, GC-MS analysis detected high levels of faecal steroids (see Table 2 SM6). Both ratios R1 and R2 had values > 0.7, pointing to a significant faecal input, according to the threshold levels proposed by Bull et al. [40] and Grimalt et al. [39]. Furthermore, R3 and R4 ascribed the faecal input to herbivores, since both ratios are lower than 0.29, as suggested by Bortolini et al. [38] and Prost et al. [74]. Very low values of Δ^5^ phytosterols (campesterol, β-sitosterol and stigmasterol) and R5 indicate digested vegetal matter in dung, as is the case here, rather than simple accumulations of bedding/fodder material.

The palynological record from the same dung-rich deposits analysed for GC-MS, i.e., most likely reflecting the diet of domestic animals, is mainly composed of pollen of wild herbs, forbs, and grasses of terrestrial open environments, with high values of specific pasture/meadows indicators and dry grasslands indicators. Sedges from humid habitats could have also been used for fodder and bedding, as did ferns. Arboreal pollen, mainly referring to deciduous mesophile woodlands, is extremely low. In structure C, pollen from cereals and from arable weeds and ruderal and nitrophilous plants is attested but less relevant compared to the other contexts of this study. Some pollen clumps and coprophilous indicators that characterise dung are present. Along the outer wall of structure C, 18 insect puparia of ‘sheep tick’ (*Melophagus ovinus*, Fig 11m) [85] were found within a small dump of pottery and bone fragments [79]. The presence of these puparia in the archaeological record is usually correlated with that of sheep and specifically with wool shearing and processing [86,87], commonly carried out in the interiors of domestic structures [88]. It is likely that the penning of sheep could have taken place in structure C for shearing activities. The absence of a signal in GC-MS and in the macrobotanical record of accumulated plant material, e.g., for fodder and bedding, suggests that likely also most of the pollen, which depicts open wild vegetation of summer pastures, entered the record with dung pellets and attached to the fur of animals. Nevertheless, we cannot exclude some plant use for food preparations, e.g., in case of pollen of cereals and goosefoot. Rare wood and bark remains over 4 cm in size are often mineralised or encrusted with sediment. The identified fragments belong to oaks and alder and have been interpreted as trampled litter material for animal bedding [79].

#### Structure G.

In structure G thin section analysis revealed that the stratigraphy is composed of ash rake-out from hearts and particularly thick layers of herbivore dung and bedding material or stored fodder. Charred macro-remains correspond to few grains and chaff of barley, emmer and einkorn, and many seeds of goosefoot - also known to be human food [89]. These most likely derive from ash rake-out. On the other hand, the uncharred seeds of a large variety of fruit plants such as hazelnuts, grape pips, blackberry, and cornelian cherry endocarps likely derive from dung or fodder, attesting the diet of animals also included many fruits from woodland edges. Uncharred small seeds, such as those of berries, can be concentrated in human faeces [90]. Nevertheless, GC-MS analysis revealed also in this structure a faecal input derived exclusively from herbivores as suggested by R3 (0.09–0.20) and R4 (0.07–0.19). An origin from human faecal input can therefore be excluded. Furthermore, biomarker analysis suggests that the faecal input in structure G is more variable among the samples (Fig 13). This data supports the observation from soil micromorphology of the presence of herbivore dung accompanied by bedding materials (indicated by the presence of Δ^5^ phytosterols). In units 646 and 654 high values of R1 and R2 (both with a mean value of 0.75) indicate dung, whereas unit 635 seems to be less affected by faecal matter (only one sample has R1 and R2 > 0.7, the others have a mean value of 0.39 and 0.50 for R1 and R2, respectively) thus suggesting the accumulation of fodder/bedding material.

A dominance of non-arboreal pollen of terrestrial open habitats, with remarkable high values of pollen indicators of pasture/meadows (13.9%), arable weeds/plants of ruderal habitats, dry grasslands, and some wet meadows indicators characterises the record of dung/bedding-rich layers in structure G. Similarly to structure C, high value of sedges pollen and some fern spores indicate further possible source of fodder or bedding. Pollen from shrubs, i.e., heliophilous and fruit-bearing plants indicating forest borders and openings, is well-represented and it supports the presence of fruits and berries seen in the macrobotanical record. In hut G there is the lowest cereal pollen value of this study. Pollen clumps and coprophilous indicators are present with low values, thus supporting the possibility of storage of vegetal matter and not only dung presence. Also, in this structure, some wood remains of oak and alder might be interpreted as bedding material for the penned animals [79].

#### Structure F.

In structure F, the stratigraphy consists of an alternating sequence of ash and trampled dung, with inclusion of waste from cereal processing and burnt animal bones derived from food preparation.

Faecal biomarkers show a similar pattern to structure C. The total steroid content is high and R1 (0.94–1) and R2 (0.80–0.95) confirm the presence of faecal matter. R3 (0.12–0.29) and R4 (0.11–0.24) indicate a faecal source related to herbivorous mammals. In ash rake-out, traces of cereal processing are indicated by numerous charred chaff and some broomcorn millet grains probably burnt during parching or roasting. As for structure E, this evidence in the macros is associated with a very high value of pollen of cereals spread in the opening of glumes and ears. Coprophilous indicators have the highest value of the study in dung-rich layers. The macrobotanical record is mainly composed of seeds of typical wetland plants, in particular numerous uncharred seeds of distant sedge (*Carex distans*). These sedges grow in wet meadows, often on sandy soil with ruderal vegetation. Sedge pollen is very abundant too. Sedges could represent locally available fodder source to be harvested in autumn-winter, when other kinds of resources were rare, or could be brought in the record by animals grazing on wetland pastures close to the site. Uncharred seeds of carrot are also frequent, a plant that has been found also as pollen. The latter in its wild state is considered to grow on pastures, and is easily found in open, sunny places. Further pollen of grasslands and pastures/meadows indicating pastureland or vegetation used for haymaking are well represented, also as frequent pollen clumps. In addition, most of the layers in the structure also contained some uncharred seeds of arable weeds, including goosefoot, St. John’s wort (*Hypericum perforatum*), annual yellow woundwort and common vervain that could also derive from fodder. In structure F, arboreal pollen is slightly more abundant, with hygrophilous woodland and high deciduous mesophile woodland and shrubs. Uncharred acorns confirm the use of woodland resources, as do wood remains. In this structure, uncharred and mineralised small wood branches, mainly of elm, may indicate winter fodder, before the flowering season.

#### Structure E.

In structure E, the sequence consists of an alternating sequence of ash and trampled dung, similarly to the other studied structures. In the ash, charred chaff remains from cereal dehusking are particularly abundant in thin section. This final stage of crop processing took place inside structures, prior to food preparation, possibly on a daily basis. From other coeval sites it is known that grains were stored as hulled spikelets or ears to be more resistant to fungal attack, e.g., full ears have been found at the early-middle bronze age pile dwelling site of Lucone (Garda Lake - [80]). This presence of chaff is attested by charred glume bases and rachises of different *Triticum* sp. in the macro-botanical record (e.g., unit 529). Also, pollen of cereals scattered from the glumes during processing [91] has here the highest value of this study. In the macrobotanical record arable weeds and barnyard grass, that could also be connected to cereal-based food preparation, are very frequent too. In units interpreted as trampled dung (e.g., unit 619), many plant species related to herbivore nutrition are attested. For instance, the meadow/grassland category typical of pastures, shows a good taxonomic richness in both pollen and macrobotanical remains, with plant taxa not attested elsewhere. Uncharred terminal bud fragments attributed to deciduous oak (after [92]) have been also recovered, indicating late winter-early spring: the vegetative development of the buds, which have not yet hatched, could coincide with a period between the end of winter and the beginning of spring (March-April). In this period, the collection of leafy hay from woodlands could provide fodder, waiting for grasslands to regenerate and be used in late spring and summer for pastures. In the pollen, the arboreal component is dominated by hygrophilous woods, attested also by uncharred wood and bark fragments of mainly alder and *Clematis vitalba*. Sedges are also very abundant in both the carpological as well as the pollen records, and they could also provide fodder all year round [93]. Coprophilous indicators support the evidence of dung.

### 4.3 What are the micromorphological characteristics of trampled herbivore dung in waterlogged conditions?

Micromorphology was key to identify herbivore dung accumulations. This was especially the case when dung was several cm thick (i.e., in structure G), and in the field could be misinterpreted as floors made with organic sediments, a known occurrence from other waterlogged contexts [94,95]. Despite the abundance of herbivore dung identified within the structures, faecal pellets in thin section are sporadic, as well as those recovered through flotation. This can be explained by the fact that the internal domestic space was heavily trampled, leading to the fragmentation of pellets and the rearrangement of the vegetal remains that compose them. These processes lead to compaction and strongly expressed horizontal orientation of the coarser components within stabling layers [48].

Even though faecal spherulites are reported to be easily leached and rarely preserved in waterlogged contexts [42,96], they are very well attested in the herbivore dung of Oppeano 4D. Their preservation can be attributed to the fast sedimentary accretion within the structures, implying rapid burial and protection from weathering (cf. [97,98]). Additionally, the circulating waters in this area are Ca-rich, as also testified by the perfect preservation of wood ash crystals in the deposits. Secondary phosphates, common in well-drained stabling deposits [48], are instead quite rare at Oppeano. They are in fact only documented in sporadic phosphate nodules and stabling crust fragments (see SMT 2d in Table 2). Sulphur, on the other hand, appears to be strongly correlated with waterlogged dung deposits, as highlighted by micro-XRF data (Fig 7f, h). This can be related to the excellent preservation of organic remains in waterlogged sites, while phosphorus release in stabling deposits typically arises from the decomposition of organic matter and dung [99]. However, further investigations are necessary to clarify the formation processes of secondary phosphates in stabling deposits [5].

### 4.4 Can we differentiate between herbivore dung and bedding/fodder material and human faecal material? What were the living conditions in the huts?

Faecal biomarker analysis helped to differentiate herbivore dung from bedding/fodder material in the Oppeano structures. The comparison with modern samples ([38]; this study) highlighted differences in the concentration of Δ^5^ phytosterols (campesterol, β-sitosterol and stigmasterol), mainly abundant in plant material, and 5β-phytostanols (5β-stigmastanol and epi-5β-stigmastanol), generally predominant in herbivore dung, especially of ruminants, among the structures. The proposed ratio R5 suggested a better distinction of the relative amount of dung and fodder, considering the proportion between 5β-stigmastanol congeners vs phytosterols/5α-stigmastanol (with the assumption that the majority of 5α-stigmastanol derives from the *in situ* microbial degradation of plant material). While in structure C a clear signal of herbivore dung was identified, in structures G and F also the evidence of bedding/fodder material was noticed. Micromorphological analysis further supports the GC-MS data. At Oppeano, herbivore dung derives from ruminants and therefore includes significant amounts of faecal spherulites. In bedding/fodder layers (cf. structure G), instead, faecal spherulites are clustered within dung aggregates, with a significant amount of vegetal material not associated with them and rare spherulites dispersed within the groundmass. It must be stressed, however, that in other archaeological contexts faecal spherulites may dissolve [42,96] or the herbivore faecal input may derive from animals that do not produce them (e.g., horse) [43,100]. Therefore, a multiproxy approach is required to determine the source(s) of faecal pollution and to reconstruct possible diagenetic processes.

Concerning the living conditions within the houses, few eggs of intestinal parasites such as *Trichuris* sp. and *Ascaris* sp., that could be also attributed to animals, are not enough to support the regular disposal of human faeces in the structures. No further indication of human excrement has been discovered in the field, through flotation, in thin section analysis, or via faecal biomarker analysis. Coprostanol is in fact the major 5β-stanol in human faeces, representing about the 60% of the total steroid content [78], while in the investigated samples it does not exceed 13% (see Table 2 in SM6). Maintenance practices aimed at keeping the domestic spaces within the structures as dry and healthy as possible are evident in the habit of spreading ashes and charcoal on the living floors. This practice, previously proposed by Nicosia et al. [3], for structures E and F, is also confirmed for the other huts studied so far, allowing us to consider it a routine part of the periodic maintenance of domestic spaces (cf. [101,102]).

### 4.5 Which plant components were used for animal husbandry? Can they inform us about the seasonality and the environmental impact of herding practices?

The botanical records from Oppeano 4D reflect a variety of natural environments spreading from woodlands and wetlands, to cultivated land, pastures, and other open areas in and surrounding the settlement (Fig 9). This diversity in the botanical macro-remains originates from different human activities that were carried out at the site and that involved plant resources, such as cereals for food production, as well as from plant material used as fodder or bedding for domestic animals, such as grassland species, ruderal and nitrophilous plants and some woodland resources. Since dung in soil thin section is usually not burnt, what is preserved through carbonization (i.e., cereals and chaff) has likely been processed by humans for food, while uncharred remains, given the considerable herbivore dung accumulation, can be mainly regarded as related to animal husbandry (Fig 9). The palynological record comes from dung-rich stabling layers only, thus reflecting either the dung content brought in the structures by animals that were on pastures daily, or hay collected and stored, to be given to the animals in winter, when certain kinds of pastures were not available, or whenever animals had to be kept close to humans for other reasons (e.g., lactation, births, shearing). The distinction between an origin of the pollen from digested vegetal matter or from hay storage is not straightforward. From other European prehistoric sites, the summer collection of green leafy hay from deciduous trees is known to be an important resource for litter and fodder in the winter months [4,67,103], to which in late winter and early spring catkins (e.g., of alder, birch, and hazel) and leaves of evergreen plants such as ivy (*Hedera*) and mistletoe (*Viscum*) could be added [68,104]. From the Po Plain, in the terramara of S.Rosa di Poviglio (Reggio Emilia), the charcoal record suggested the use of elm for leafy hay [105], whereas in the terramara of Montale (Modena) accumulation of hornbeam pollen in the archaeological stratigraphy has been associated to forage [106,107]. In Oppeano 4D there are some plant indicators of this practice, as evidenced by the discovery of some unburnt terminal buds of *Quercus* cf. *robur* in structure E, some alder pollen clumps, and some uncharred elm twigs in structure F. However, from the pollen record of the stabling layers, the relevance of arboreal resources for animal herding seems to have played a minor role compared to the variety of plants reflecting the vegetation of open environments, common also the carpological record. Humid open areas dominated by sedges (Cyperaceae, *Carex*), which are abundant and ubiquitous in both the carpological and the pollen records, were collected or grazed as fodder and could be found in minor spring-fed rivers near the site. In structure F, a particularly abundant accumulation of uncharred sedge seeds (*Carex distans*) and pollen is found, together with carrot seeds (*Daucus carota*), suggesting a provenance from wet meadows, further supported also by pollen of wetland species (e.g., *Lythrum salicaria*). Sedges are not usually considered as high quality fodder [108], but are widely used for that (e.g., in the hay management of wetlands) [93,109] and for other purposes such as matting and thatching. Moreover, they offered green foliage for pasture and hay all year round [93,110]. Another kind of environment used for pastures is represented by the xerophytic vegetation of dry grasslands, which likely could grow on sandy soils of the LGM Adige alluvial fan. In particular, the ubiquitous presence in huts C and G of the pollen of *Helianthemum* sp., a small shrub with no nutritious value [108], likely selected by the herbivores for medical properties (as attested today for wild ungulates) [111], suggests that these environments were used for pastures more than for hay collection. Plants of this environment are blooming in summer and could indicate pasturelands on drained calcareous land. The development of these secondary grasslands seems to be enhanced by MBA grazing pressure [61,112]. A similar spread of pastureland has been proposed for other sites of the Po Plain[113], supported by the increase of Cichorieae pollen [114], whose high values have been observed also in phosphatic crusts derived from herbivore dung at the site of La Muraiola (Verona) [5]. This indicator of pastures in Oppeano 4D is regularly present, although with low relative values due to the variety and abundance of other *taxa*. Cereals did not contribute to animal diet for livestock, because grains and chaff in the carpological record are charred (Fig 9). The high amount of cereal pollen derives from dehusking, the routine activity that took place prior to food preparation inside the huts and that resulted in the charring of chaff as leftovers (e.g., in structure E). However, we cannot say the same for arable weeds, some of which are charred, such as barnyard grass (which is perhaps also considerable as a millet cereal itself) and goosefoot, and other uncharred seeds/fruits of grassland and wetland species, trees and shrubs. Since biomarker analysis excludes human dejections, uncharred seeds are likely to derive from dung: in structure G and E, seeds of bramble and cornelian cherry were found that could be ingested by animals, although fruits were collected most likely also for human diet. Cornelian cherry was a common fruit in the northern Italian Bronze Age and sometimes concentrations of *Cornus mas* stones have been found that suggested some form of processing into a (possibly fermented) beverage [115,116]. Arable weeds and plants of ruderal and nitrophilous habitats are extremely abundant and diverse in the pollen record too. This frequency and high diversity indicate that marginal areas around the settlement could be used as pastures and for the collection of hay too, as known in historical times [117]. Based on the presence of weeds, it is possible also that arable plots for cereal agriculture were used themselves for pasture after harvest (late summer/early autumn). In structure E, many of these species are attested in both the botanical macro-remains and the pollen records (e.g., *Potentilla*, *Agrimonia*, Amaranthaceae), along with other indicators of pastures and meadows (e.g., Fabaceae such as *Medicago* in the carpological record and *Trifolium* in the pollen record, where there are also *Centaurea nigra* type, *Plantago lanceolata*, *Ranunculus acris* type, Cichorieae, *Stachys*, *Helianthemum*).

Concerning seasonality, the wide spectrum of plant resources suggests that the site was occupied all year round and that people had a sustainable use of local environments and vegetation, spanning from wet to dry habitats, forests, and grasslands. The occurrence of such a wide spectrum of different herbs and grasses compared to the little evidence from trees and shrubs (many of which produce pollen and buds in late winter/early spring) suggests that in winter the animals were fed with hay fodder mainly from herbs and grasses besides some tree catkins and twigs. Hay was collected possibly in late summer, when seeds of carrots and sedges are ripened, and most of the dry grasslands and pasture/meadows species are blossoming, to be stored for the winter months or for the time when animals were kept inside. Summer pastures were available in a mosaic of alluvial environments including sandy ridges, woodland edges, and wetlands, where sedges could be used as an additional local fodder. A further indication of year-round use of the structures is given by the attestation of cereal processing, which fits the picture of a site where cereals were cultivated in a wide range of species, and then stored as hulled grains, and processed little by little at daily need (cf. [118]).

## 5. Conclusions

This study suggests that the living conditions within the Oppeano structures saw the coexistence of humans and small herbivores - most likely sheep and goat - within the domestic space. In these byre-houses, the periodic spreading of ash on living surfaces for hygienic purposes was a very well-attested practice, as was the processing of cereals which were periodically dehusked and possibly roasted by means of fire.

The identification of stabling layers was made possible by the following set of features: faecal spherulites, extremely low/absence of mineral components of the deposit, coprophilous fungal spores, eggs of nematodes/intestinal parasites, high pollen concentration (>100,000 pg/g), pollen clumps, seeds of wild plants, faecal steroids with related ratios. These, and especially R5, are also key to differentiate dung from bedding or fodder material, a distinction that cannot be made by micromorphology alone. Palynological analysis of dung layers, integrated by waterlogged botanical macroremains, allowed us to understand that plant resources from herding came from different open environments among which pastures on well-drained ground and sedge-dominated wetlands. Mesophilous and riparian woodlands also provided fodder. The high amount of summer-blossoming herbaceous species inside the studied structures might suggest the practice of haymaking. The community of herders and farmers made a wise use of the resources from the landscape surrounding the site, which allowed for settling all year round. The large variety of open environments suggests the possibility to bring the livestock to graze in pastures in spring and summer. Conversely, the animals were likely kept indoors during winter and fed with leafy hay, tall grasses, and herbs. These were probably mowed during the summer and stored, although no direct evidence of storage was found. The charred archaeobotanical record showed that a wide variety of cereals (emmer, einkorn, free-threshing wheats, new glume wheat, barley) were cultivated and processed with the use of fire inside the structures. Among the millets known for the period, broomcorn and foxtail millet are rare, whereas only barnyard grass is very common here. This plant can be considered as locally available in the wild, and the marked use of wild plant resources including edible weeds, fruits from trees and shrubs is characteristic of the site.

In socio-economic terms, the Oppeano byre-houses can be interpreted as single production units where small-scale herding and food preparation based on both cultivated and wild plant resources took place. The structures exhibit a low level of specialisation, with the same activities taking place in all of them. Ownership of livestock was possibly fragmented, with each household managing their own animals, and played a key role in the site’s subsistence economy.

## Supporting information

SM1 Micromorphology Structure E Fig 1. SM1 Stratigraphic sequence inside structure E of Oppeano 4D: thin sections from the micromorphology block 82, see Nicosia et al. [3] for complete description and study. On the right: interpretation of the thin sections, with a SMT assigned to each sub-unit and the location of subsamples for pollen analysis. Fig 2. SM1 Stratigraphic sequence inside structure E of Oppeano 4D: thin sections from the micromorphology blocks 80-81, see Nicosia et al. [3] for complete description and study. On the right: interpretation of the thin sections, with a SMT assigned to each sub-unit and the location of subsamples for pollen analysis.
(DOCX)

SM2 Micromorphology Structure F Stratigraphic sequence inside structure F of Oppeano 4D: thin sections 75–78 analysed for soil micromorphology, see Nicosia et al. [3] for complete description and study. Below: interpretation of the thin sections, with a SMT assigned to each sub-unit and the location of subsamples for pollen analysis. (DOCX)

SM3 Micromorphology Structure G Thin section 100 (structure G) scan, micromorphological interpretation and micro-XRF maps. (a) PPL scan; (b) XPL scan; (c) interpretation of the thin section; (d-i) micro-XRF maps showing the abundance of specific elements. When multiple elements are displayed on the same map, the resulting colour is a combination of the individual colours of each element. Note that the highest concentrations of Ca and P are visible in the stable crust fragments (arrow in ‘a’). Otherwise, in general, the P value is nearly absent.
(DOCX)

SM4 Macro-remains Table 1. SM4 Macro-remains dataset by unit of Oppeano 4D, structures C, G, D, and F. Table 2. SM4 Carpological record subdivided by structure and category.
(DOCX)

SM5 Palynology Pollen dataset of the study of Oppeano 4D structures C, G, E, F: counts and percentage values. (XLSX)

SM 6 Biomarkers Table 1. SM6 Acronyms, common and IUPAC names of the investigated steroids and the selected internal standard with the related m/z ions used for GC-MS analysis. Table 2. SM6 Steroids concentrations (ng/g) in the 24 samples from structures C, G and F of Oppeano site. COP = Coprostanol; EPI-COP = Epi-coprostanol; CHL = Cholesterol; CHN = 5α-cholestanol; CHONE = Cholestanone; 24-COP = 5β-stigmastanol; 24-EPI-COP = Epi-5β-stigmastanol; CAMP = Campesterol; STGR = Stigmasterol; β-SIT = β-sitosterol; STGN = 5α-stigmastanol. < MDL = lower than the method detection limit. Table 3. SM6 Steroids concentrations (µg/g) of crops and leaves of trees representative of the Bronze Age period. COP = Coprostanol; EPI-COP = Epi-coprostanol; CHL = Cholesterol; CHN = 5α-cholestanol; CHONE = Cholestanone; 24-COP = 5β-stigmastanol; 24-EPI-COP = Epi-5β-stigmastanol; CAMP = Campesterol; STGR = Stigmasterol; ERGO = Ergosterol; β-SIT = β-sitosterol; STGN = 5α-stigmastanol.
(DOCX)
