# Supplementary material for: Multiproxy analysis of stabling layers in four middle bronze age byre-houses from the site of Oppeano 4D (Verona, Italy)
Source: PLoS One. 2025 May 22;20(5):e0323724. doi: 10.1371/journal.pone.0323724 (PMC12097577; doi:10.1371/journal.pone.0323724)
Supplement: SM1 Micromorphology Structure E — Fig 1. SM1 Stratigraphic sequence inside structure E of Oppeano 4D: thin sections from the micromorphology block 82, see Nicosia et al. [3] for complete description and study. On the right: interpretation of the thin sections, with a SMT assigned to each sub-unit and the location of subsamples for pollen analysis. Fig 2. SM1 Stratigraphic sequence inside structure E of Oppeano 4D: thin sections from the micromorphology blocks 80-81, see Nicosia et al. [3] for complete description and study. On the right: interpretation of the thin sections, with a SMT assigned to each sub-unit and the location of subsamples for pollen analysis. (DOCX) [file pone.0323724.s001.docx]

**Supplementary Material 1 Micromorphology Structure E** of the manuscript Nicosia et al.


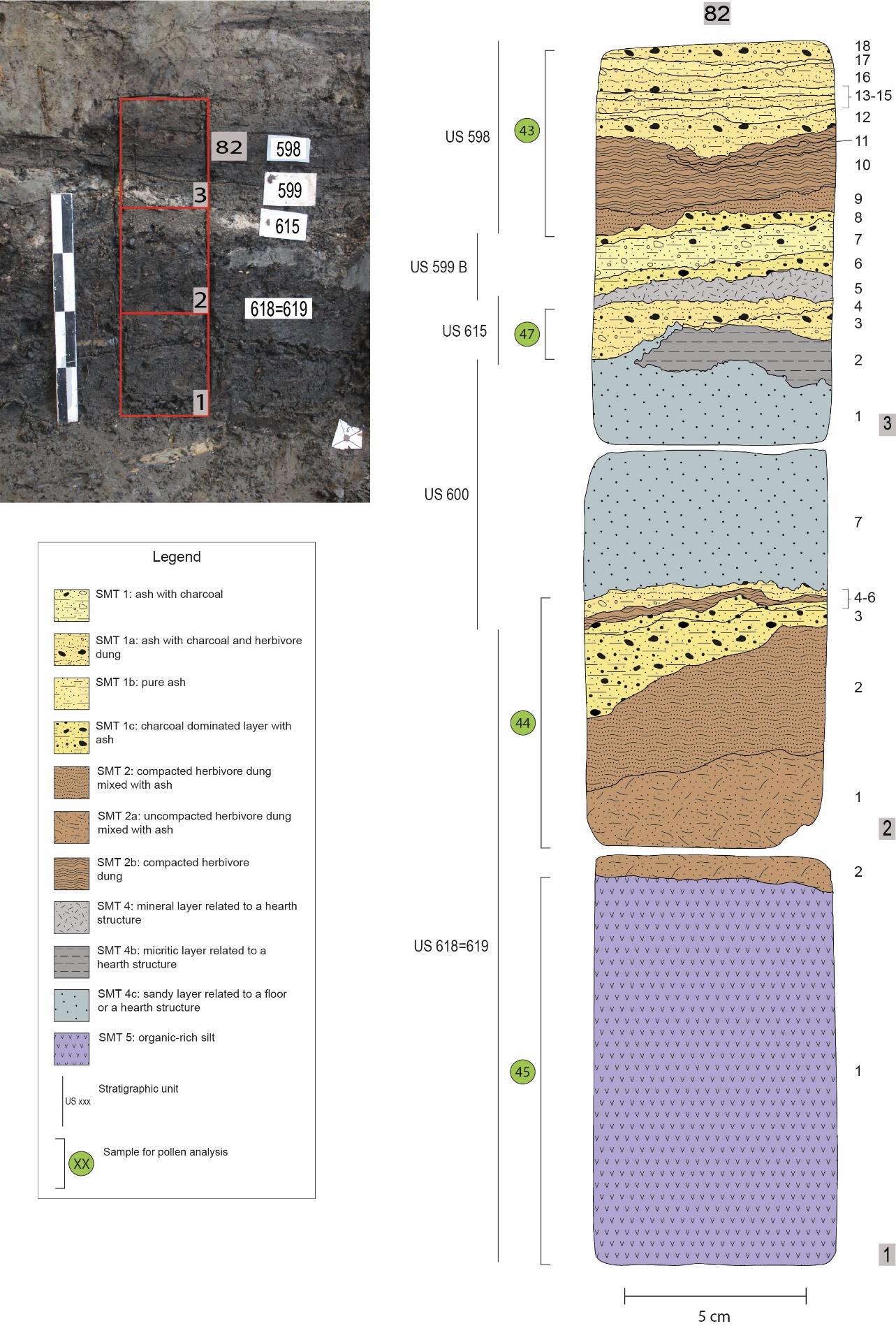


**Fig. 1 SM1** Stratigraphic sequence inside structure E of Oppeano 4D: thin sections from the micromorphology block 82, see Nicosia et al. (2022) for complete description and study. On the right: interpretation of the thin sections, with a SMT assigned to each sub-unit and the location of subsamples for pollen analysis.


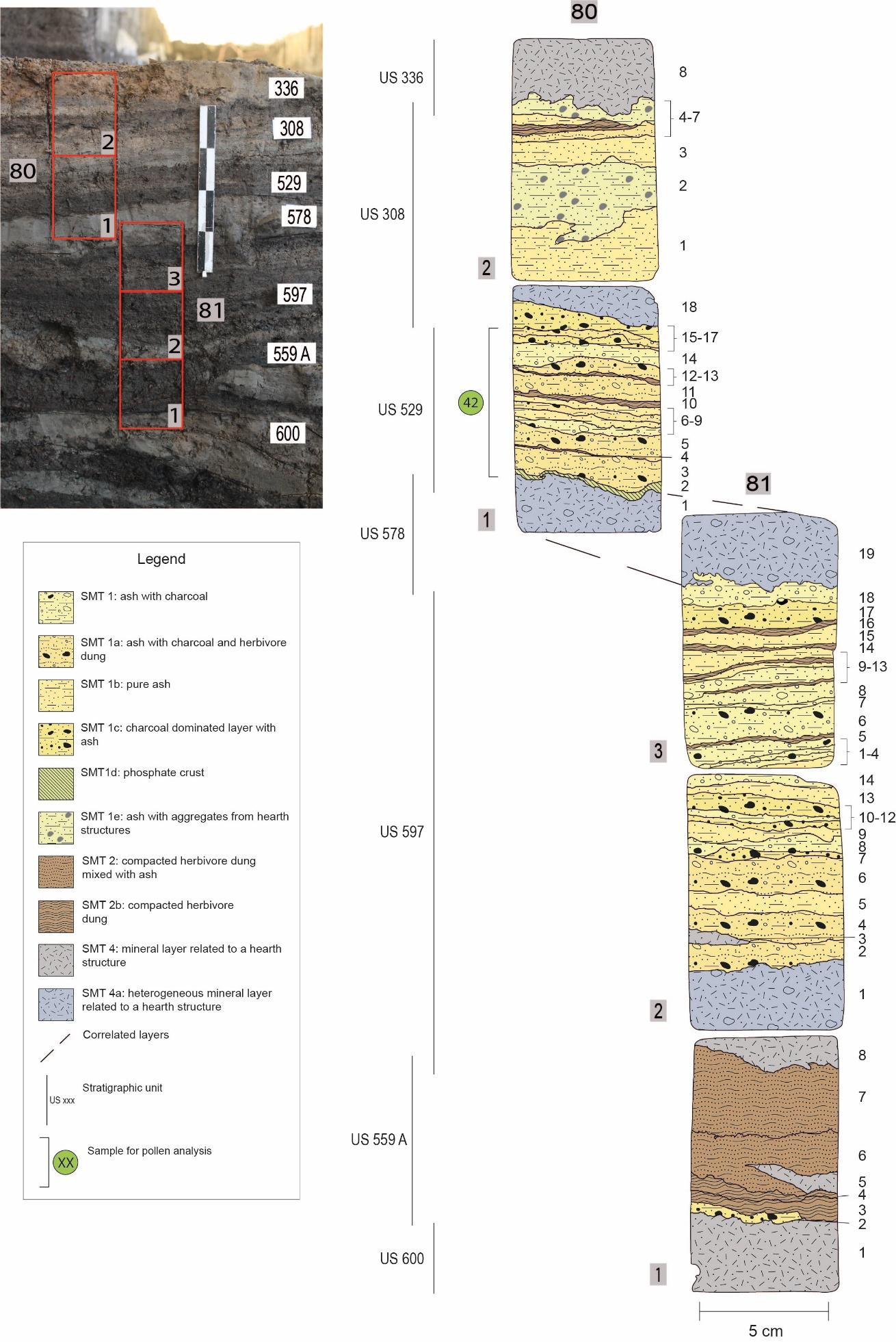


**Fig. 2 SM1** Stratigraphic sequence inside structure E of Oppeano 4D: thin sections from the micromorphology blocks 80-81, see Nicosia et al. (2022) for complete description and study. On the right: interpretation of the thin sections, with a SMT assigned to each sub-unit and the location of subsamples for pollen analysis.
