# Supplementary material for: Multiproxy analysis of stabling layers in four middle bronze age byre-houses from the site of Oppeano 4D (Verona, Italy)
Source: PLoS One. 2025 May 22;20(5):e0323724. doi: 10.1371/journal.pone.0323724 (PMC12097577; doi:10.1371/journal.pone.0323724)
Supplement: SM2 Micromorphology Structure F — (DOCX) [file pone.0323724.s002.docx]

**Supplementary Material 2 Micromorphology Structure F** of the manuscript Nicosia et al.


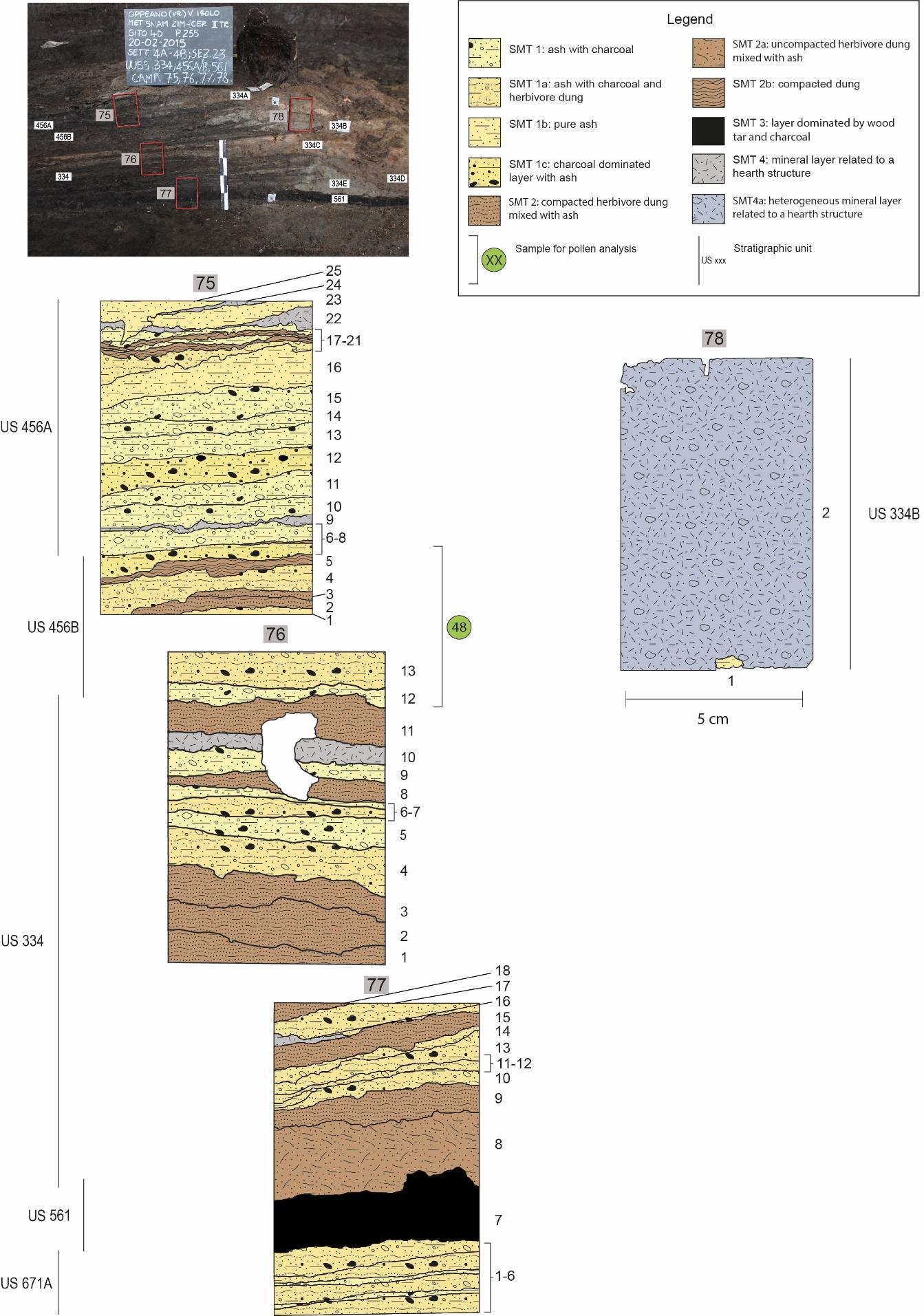


Stratigraphic sequence inside structure F of Oppeano 4D: thin sections 75-78 analysed for soil micromorphology, see Nicosia et al. (2022) for complete description and study. Below: interpretation of the thin sections, with a SMT assigned to each sub-unit and the location of subsamples for pollen analysis.
