# Supplementary material for: Multiproxy analysis of stabling layers in four middle bronze age byre-houses from the site of Oppeano 4D (Verona, Italy)
Source: PLoS One. 2025 May 22;20(5):e0323724. doi: 10.1371/journal.pone.0323724 (PMC12097577; doi:10.1371/journal.pone.0323724)
Supplement: SM3 Micromorphology Structure G — Thin section 100 (structure G) scan, micromorphological interpretation and micro-XRF maps. (a) PPL scan; (b) XPL scan; (c) interpretation of the thin section; (d-i) micro-XRF maps showing the abundance of specific elements. When multiple elements are displayed on the same map, the resulting colour is a combination of the individual colours of each element. Note that the highest concentrations of Ca and P are visible in the stable crust fragments (arrow in ‘a’). Otherwise, in general, the P value is nearly absent. (DOCX) [file pone.0323724.s003.docx]

**Supplementary Material 3_Micromorphology Structure G** of the manuscript Nicosia et al.


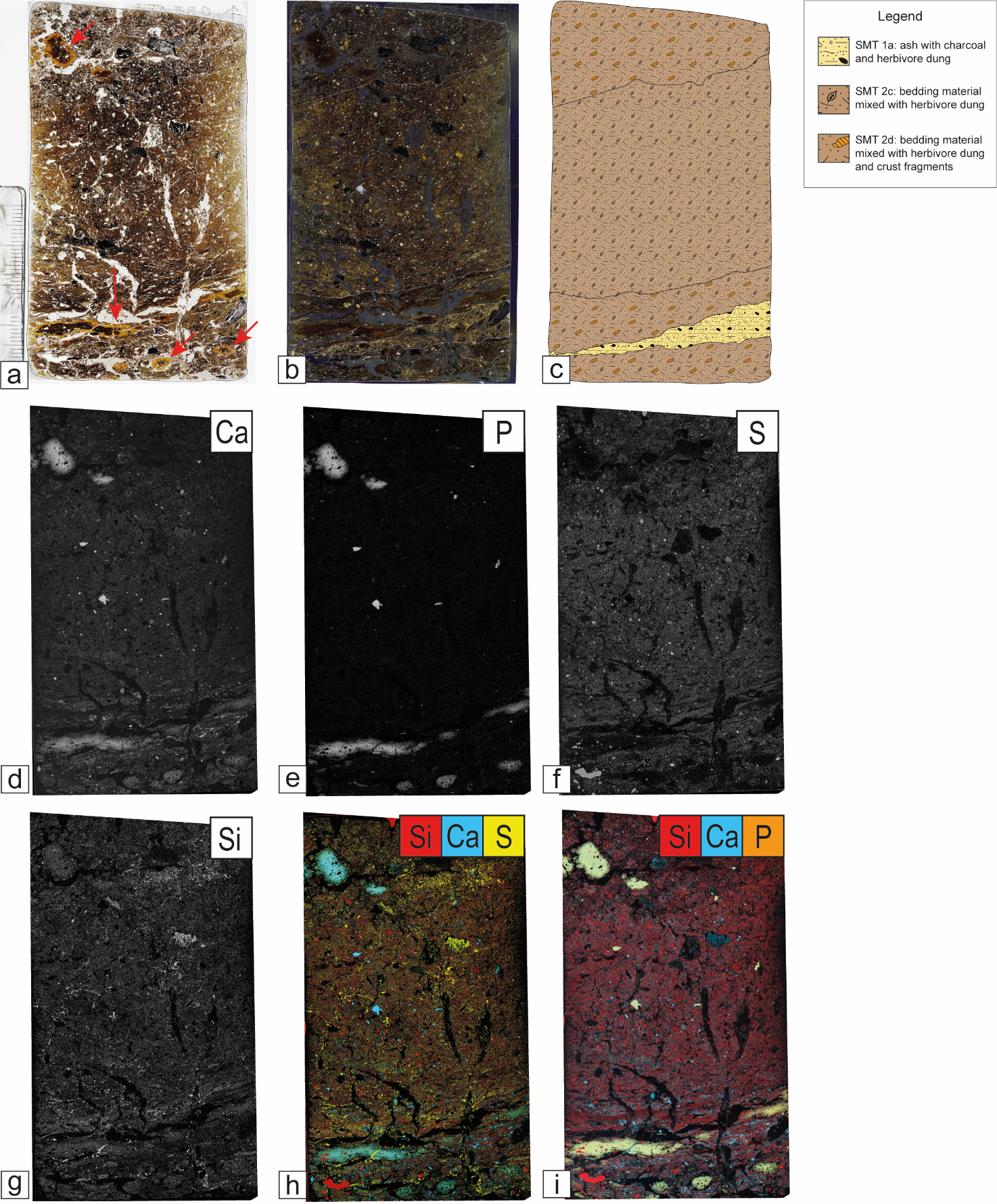


Thin section 100 (structure G) scan, micromorphological interpretation and micro-XRF maps. a) PPL scan; b) XPL scan; c) interpretation of the thin section; d-i) micro-XRF maps showing the abundance of specific elements. When multiple elements are displayed on the same map, the resulting colour is a combination of the individual colours of each element. Note that the highest concentrations of Ca and P are visible in the stable crust fragments (arrow in ‘a’). Otherwise, in general, the P value is nearly absent.
