# Supplementary material for: Multiproxy analysis of stabling layers in four middle bronze age byre-houses from the site of Oppeano 4D (Verona, Italy)
Source: PLoS One. 2025 May 22;20(5):e0323724. doi: 10.1371/journal.pone.0323724 (PMC12097577; doi:10.1371/journal.pone.0323724)
Supplement: SM4 Macro-remains — Table 1. SM4 Macro-remains dataset by unit of Oppeano 4D, structures C, G, D, and F. Table 2. SM4 Carpological record subdivided by structure and category. (DOCX) [file pone.0323724.s004.docx]

**Supplementary Material 4** of the manuscript Nicosia et al.

**Tab. 1 SM4** Macro-remains dataset by unit of Oppeano 4D, structures C, G, D, and F

| **Structures** | | | **C** | | **G** | | | | **E** | | | | **F** | | | | | | | | | **TOT.** |
| --- | --- | --- | --- | --- | --- | --- | --- | --- | --- | --- | --- | --- | --- | --- | --- | --- | --- | --- | --- | --- | --- | --- |
| **Context** | | | **SU 415C** | **SU 382** | **SU 658** | **SU 635** | **SU 654** | **SU 679** | **SU 619** | **SU 578** | **SU 308** | **SU 529** | **SU 594** | **SU 606** | **SU 608** | **SU 632** | **SU 587** | **SU 456** | **SU 563** | **SU 555** | **SU 585** |  |
| **Sample volume/L** | | | **1,09** | **0,6** | **0,68** | **0,15** | **0,55** | **0,35** | **0,45** | **0,6** | **0,86** | **2,06** | **0,7** | **0,67** | **0,35** | **0,53** | **0,6** | **0,54** | **0,5** | **0,4** | **0,4** | **12,08** |
| **Categories** | **Plant part** | **Cons.** |  |  |  |  |  |  |  |  |  |  |  |  |  |  |  |  |  |  |  |  |
| **Cereals** |  |  |  |  |  |  |  |  |  |  |  |  |  |  |  |  |  |  |  |  |  |  |
| *Hordeum vulgare/distichum* | grain | c | 1 | 2 | 2 |  |  |  |  |  |  |  |  |  |  | 2 |  | 2 |  |  |  | **9** |
| *Panicum miliaceum* | grain | c |  | 2 |  |  |  |  |  |  |  |  |  |  |  |  |  | 2 | 1 |  |  | **5** |
| *Setaria italica* | grain | c |  | 1 |  |  |  | 1 | 1 |  |  |  |  |  |  |  |  |  |  |  |  | **3** |
| *Triticum aestivum/durum* | grain | c |  |  |  |  |  |  |  |  |  | 3 |  |  |  | 1 |  | 1 |  |  |  | **5** |
| *Triticum aestivum/durum* | rachis | c |  |  |  |  |  |  | 3 |  |  | 4 |  |  |  | 1 |  | 3 |  |  |  | **11** |
| *Triticum dicoccum* | grain | c | 1 |  | 1 |  | 1 |  |  |  |  | 5 |  |  |  | 3 |  | 1 | 1 |  |  | **13** |
| *Triticum dicoccum* | glume base | c | 2 |  |  |  | 1 |  | 1 |  |  | 1 |  |  |  | 1 |  | 4 | 4 | 1 |  | **15** |
| *Triticum dicoccum/timopheevii* | glume base | c |  |  | 2 |  |  |  |  |  |  | 11 |  |  |  |  | 1 | 1 |  |  |  | **15** |
| *Triticum timopheevii* | glume base | c | 1 |  |  |  |  |  |  |  |  |  |  |  |  |  |  |  |  |  | 1 | **2** |
| *Triticum monococcum* | grain | c |  |  | 1 |  |  |  |  |  |  | 2 |  |  |  |  |  |  | 1 |  |  | **4** |
| *Triticum monococcum* | glume base | c | 2 | 5 | 6 |  |  | 5 |  | 8 | 2 | 25 | 5 |  | 5 | 2 | 5 | 9 | 3 | 1 |  | **83** |
| *Triticum monococcum* | glume base | uc |  |  |  | 1 |  |  | 1 |  |  |  |  |  |  |  |  |  |  |  |  | **2** |
| *Triticum* sp. | grain | c |  | 1 |  |  |  |  |  |  |  | 4 |  |  |  |  |  |  |  |  |  | **5** |
| *Triticum* sp. | glume base | c |  | 2 |  |  |  | 1 |  |  | 2 | 45 |  |  |  |  | 3 | 4 | 2 |  |  | **59** |
| *Triticum* sp. | culm | uc |  |  | 2 |  |  |  |  |  |  |  |  |  |  |  |  |  |  |  |  | **2** |
| **Pulses** |  |  |  |  |  |  |  |  |  |  |  |  |  |  |  |  |  |  |  |  |  |  |
| *Pisum* sp. | seed | c | 1 |  |  |  |  |  |  |  |  |  |  |  |  |  |  |  |  |  |  | **1** |
| *Vicia* cf. *sativa* | seed | uc |  | 5 |  |  |  |  |  |  |  |  |  |  |  |  |  |  |  |  |  | **5** |
| **Flax** |  |  |  |  |  |  |  |  |  |  |  |  |  |  |  |  |  |  |  |  |  |  |
| *Linum usitatissimum* | seed | c |  | 17 |  |  |  |  |  |  |  |  | 1 |  |  | 2 | 1 |  |  |  |  | **21** |
| *Linum usitatissimum* | boll | uc |  |  |  |  |  |  |  |  |  |  |  | 2 |  |  |  |  |  |  |  | **2** |
| **Arable weeds (Echinochloa)** |  |  |  |  |  |  |  |  |  |  |  |  |  |  |  |  |  |  |  |  |  |  |
| *Echinochloa crus-galli* | grain | c | 85 | 41 | 3 | 6 | 7 | 14 | 1 | 6 | 10 | 26 | 7 | 1 | 4 | 7 | 1 | 36 | 4 |  | 8 | **267** |
| *Echinochloa crus-galli* | grain | uc |  |  |  |  |  |  |  |  |  |  |  |  |  | 12 |  | 12 |  |  |  | **24** |
| **Arable weeds, ruderal and nitrophilous plants** | |  |  |  |  |  |  |  |  |  |  |  |  |  |  |  |  |  |  |  |  |  |
| *Avena* sp. | grain | c |  |  |  |  | 1 |  |  |  |  | 1 |  |  |  |  |  |  |  |  |  | **2** |
| *Chenopodium album* | seed | c | 1 | 13 |  | 2 | 1 | 65 | 1 |  |  |  |  |  |  |  |  |  |  |  |  | **83** |
| *Chenopodium album* | seed | uc |  |  |  |  |  |  |  |  |  | 1 | 9 |  |  | 8 | 1 | 2 | 3 |  |  | **24** |
| *Chenopodium ficifolium* | seed | uc |  |  |  |  |  |  |  |  |  | 3 |  |  |  |  |  |  |  |  |  | **3** |
| *Daucus carota* | seed | uc |  |  |  |  |  |  | 1 |  |  |  | 1 | 29 | 1 | 1 |  |  |  |  | 3 | **36** |
| *Fallopia convolvulus* | seed | c |  |  | 6 |  |  |  |  |  |  |  |  |  |  |  |  | 2 |  |  |  | **8** |
| *Fallopia convolvulus* | seed | uc |  |  |  |  |  |  | 8 |  |  |  |  |  |  |  |  |  |  |  |  | **8** |
| *Galeopsis* cf. *tetrahit* | seed | uc |  |  |  |  | 1 |  |  |  |  |  |  |  |  |  |  |  |  |  |  | **1** |
| *Galium aparine* | seed | c |  |  |  |  |  |  |  |  |  |  |  |  |  | 4 |  |  | 1 |  |  | **5** |
| *Galium* sp. | seed | uc |  |  |  |  |  |  |  |  |  |  |  |  | 1 |  |  |  |  |  |  | **1** |
| *Hypericum perforatum* | seed | uc |  |  |  |  |  |  |  |  |  |  | 5 |  |  |  |  |  |  |  |  | **5** |
| *Persicaria maculosa* | seed | uc |  |  | 1 |  |  |  |  |  |  |  |  |  |  |  |  |  |  |  | 4 | **5** |
| *Portulaca oleracea* | seed | c |  | 1 |  |  |  |  |  |  |  |  |  |  |  |  |  |  |  |  |  | **1** |
| *Portulaca oleracea* | seed | uc |  |  |  | 1 |  |  |  |  |  |  | 2 |  |  |  | 1 |  |  |  |  | **4** |
| *Silene latifolia* | seed | uc |  |  |  |  |  |  |  |  | 2 |  |  |  |  |  |  |  |  |  |  | **2** |
| *Solanum nigrum* | seed | c |  | 1 |  |  |  |  |  |  |  |  |  |  |  |  |  |  |  |  |  | **1** |
| *Stachys annua* | seed | uc |  |  |  |  | 3 |  |  |  |  |  | 2 |  |  |  |  |  |  |  |  | **5** |
| *Verbena officinalis* | seed | uc |  |  |  |  |  |  |  |  |  |  | 6 |  |  |  |  |  |  |  |  | **6** |
| *Urtica dioica* | seed | uc |  |  |  |  |  |  | 4 |  |  | 2 |  |  |  |  |  |  |  |  |  | **6** |
| **Grassland plants** |  |  |  |  |  |  |  |  |  |  |  |  |  |  |  |  |  |  |  |  |  |  |
| *Agrimonia eupatoria* | seed | c |  |  |  |  |  | 1 |  |  |  |  |  |  |  |  |  |  |  |  |  | **1** |
| *Agrimonia eupatoria* | seed | uc |  |  |  |  |  |  | 2 |  |  |  |  |  |  |  | 1 |  |  |  |  | **3** |
| *Ajuga chamaepitys* | seed | c |  |  |  |  |  |  |  |  |  |  |  |  |  |  |  |  | 2 |  |  | **2** |
| *Crupina* cf. *vulgaris* | seed | uc |  |  |  |  |  | 1 | 3 |  |  |  |  |  |  |  |  |  |  |  |  | **4** |
| *Fragaria vesca* | seed | uc |  | 2 |  |  |  |  | 1 |  |  |  |  |  |  |  |  |  |  |  |  | **3** |
| *Medicago* cf. *polymorpha* | seed | uc |  |  |  |  |  |  | 1 |  |  |  |  |  | 1 | 1 |  |  |  |  |  | **3** |
| *Potentilla argentea* | seed | uc |  |  |  |  |  |  |  |  |  |  |  |  |  |  |  |  |  |  | 4 | **4** |
| *Potentilla* sp. | seed | uc |  |  |  |  |  |  | 7 |  |  |  |  |  |  |  |  | 1 |  |  |  | **8** |
| *Ranunculus acris* | seed | c |  |  |  |  |  |  |  |  |  |  |  | 3 |  |  |  |  |  |  |  | **3** |
| *Ranunculus acris* | seed | uc |  |  |  | 1 | 2 |  |  |  |  |  |  |  |  |  |  |  |  |  |  | **3** |
| *Valerianella dentata* | seed | uc |  |  |  |  |  |  |  |  |  |  | 4 |  |  |  |  |  |  |  |  | **4** |
| *Valerianella* sp. | seed | uc |  |  |  |  |  |  |  |  |  | 1 |  |  |  |  |  |  |  |  |  | **1** |
| **Wetland plants** |  |  |  |  |  |  |  |  |  |  |  |  |  |  |  |  |  |  |  |  |  |  |
| *Carex* cf. *acuta* | seed | uc |  |  |  |  |  |  |  |  |  |  |  | 24 |  |  |  |  |  |  |  | **24** |
| *Carex* cf. *acutiformis* | seed | uc |  |  |  |  |  |  |  |  |  |  |  |  | 6 |  |  |  |  |  | 10 | **16** |
| *Carex distans* | seed | c |  |  |  |  |  | 4 |  |  |  |  |  |  |  |  |  |  |  |  |  | **4** |
| *Carex distans* | seed | uc |  | 2 |  | 1 |  |  |  |  |  |  |  | 613 | 3 |  |  |  |  |  |  | **619** |
| *Carex* cf. *flava* | seed | c |  | 4 |  |  |  |  |  |  |  |  |  |  |  |  |  |  |  |  |  | **4** |
| *Carex* cf*. flava* | seed | uc |  | 1 |  |  |  |  |  |  |  |  |  |  |  |  |  |  |  |  |  | **1** |
| *Carex hirta* | seed | c | 1 | 1 |  |  |  |  |  |  |  |  |  |  |  |  |  |  |  |  |  | **2** |
| *Carex hirta* | seed | uc |  |  |  |  |  |  | 1 |  | 1 |  |  |  |  |  |  |  |  |  | 2 | **4** |
| *Carex* sp. | seed | c | 1 | 2 |  |  |  |  |  |  |  |  |  |  |  |  |  |  |  |  |  | **3** |
| *Carex* sp. | seed | uc |  | 2 | 1 |  | 2 |  | 14 | 4 | 2 | 6 | 26 | 4 |  | 5 |  | 3 | 2 | 1 |  | **72** |
| *Lycopus europaeus* | seed | uc |  |  |  |  |  |  |  |  |  |  | 2 |  |  | 2 |  |  |  |  | 5 | **9** |
| *Persicaria hydropiper* | seed | uc |  |  |  |  |  | 2 |  |  |  |  |  |  |  |  |  |  |  |  |  | **2** |
| *Persicaria lapathifolia* | seed | c |  |  |  |  |  |  |  |  |  |  | 1 |  |  |  |  |  |  |  |  | **1** |
| *Persicaria lapathifolia* | seed | uc |  |  |  |  |  |  |  |  |  |  |  |  | 1 |  |  |  |  |  |  | **1** |
| *Ranunculus repens* | seed | c |  | 2 |  |  |  |  |  |  |  |  |  |  |  |  |  |  |  |  |  | **2** |
| *Ranunculus sardous* | seed | c |  |  |  |  |  |  |  |  |  |  |  |  |  |  |  |  | 1 |  |  | **1** |
| *Ranunculus sardous* | seed | uc |  |  |  |  |  |  |  |  |  | 1 |  |  |  |  |  |  |  |  |  | **1** |
| *Scirpus* sp. | seed | uc |  |  |  |  |  |  |  |  |  |  |  |  | 1 |  |  |  |  |  |  | **1** |
| *Stachys* cf*. palustris* | seed | uc |  |  |  |  |  | 1 |  |  |  |  |  | 13 |  |  |  |  |  |  |  | **14** |
| **Trees and Shrubs (fruits, nuts and buds)** | |  |  |  |  |  |  |  |  |  |  |  |  |  |  |  |  |  |  |  |  |  |
| *Cornus mas* | seed | c |  |  |  |  |  |  |  |  |  |  |  |  |  |  |  |  | 2 |  |  | **2** |
| *Cornus mas* | seed | uc | 1 | 3 |  |  | 3 |  | 8 |  |  |  | 1 |  |  |  |  |  |  |  |  | **16** |
| *Corylus avellana* | shell | uc |  |  | 4 |  | 3 | 13 | 1 | 3 |  |  |  |  |  |  |  |  |  |  |  | **24** |
| *Pyrus sp.* | seed | c |  |  |  |  |  |  |  |  | 1 |  |  |  |  |  |  |  |  |  |  | **1** |
| *Pyrus/malus* | seed | uc |  |  | 1 |  |  |  |  |  |  |  |  |  |  |  |  |  |  |  |  | **1** |
| *Quercus* cf. *robur* | bud | uc |  |  |  |  |  | 1 | 3 |  |  |  |  |  |  |  |  |  |  |  |  | **4** |
| *Quercus* sp. | seed | c |  |  |  |  |  | 2 |  |  |  |  |  |  |  |  |  |  |  |  |  | **2** |
| *Quercus* sp. | acorn base | c | 1 | 3 |  |  |  |  |  |  |  |  |  |  |  |  |  |  |  |  |  | **4** |
| *Quercus* sp. | acorn base | uc |  | 1 |  |  |  |  |  |  |  |  |  |  | 11 |  |  |  |  |  |  | **12** |
| *Rubus* *fruticosus* | seed | uc |  | 1 | 1 |  | 14 |  | 8 |  |  | 5 | 4 |  |  | 1 |  |  | 2 |  |  | **36** |
| *Rubus* sp. | seed | uc |  |  | 1 |  |  |  | 2 |  |  |  | 2 |  |  | 3 |  |  |  |  | 1 | **9** |
| *Vitis vinifera* ssp*. sylvestris* | seed | uc |  |  | 5 |  | 5 |  | 3 |  |  | 2 | 1 | 1 |  | 1 |  |  |  |  | 1 | **19** |
| **Tot.** |  |  | **98** | **115** | **37** | **12** | **44** | **111** | **75** | **21** | **20** | **148** | **79** | **690** | **34** | **57** | **14** | **83** | **29** | **3** | **39** | **1709** |

**Tab. 2 SM4** Carpological record subdivided by structure and category.

| **Structures** | | | **C** | **G** | **E** | **F** | **TOT.** |
| --- | --- | --- | --- | --- | --- | --- | --- |
| **Sample volume/L** | | | **1,69** | **1,73** | **3,97** | **4,69** | **12,08** |
| **Categories** | **Plant part** | **Cons.** |  |  |  |  |  |
| **Cereals** |  |  |  |  |  |  |  |
| *Hordeum vulgare/distichum* | grain | c | 3 | 2 |  | 4 | **9** |
| *Panicum miliaceum* | grain | c | 2 |  |  | 3 | **5** |
| *Setaria italica* | grain | c | 1 | 1 | 1 |  | **3** |
| *Triticum aestivum/durum* | grain | c |  |  | 3 | 2 | **5** |
| *Triticum aestivum/durum* | rachis | c |  |  | 7 | 4 | **11** |
| *Triticum dicoccum* | grain | c | 1 | 2 | 5 | 5 | **13** |
| *Triticum dicoccum* | glume base | c | 2 | 1 | 2 | 10 | **15** |
| *Triticum dicoccum/timopheevii* | glume base | c |  | 2 | 11 | 2 | **15** |
| *Triticum timopheevii* | glume base | c | 1 |  |  | 1 | **2** |
| *Triticum monococcum* | grain | c |  | 1 | 2 | 1 | **4** |
| *Triticum monococcum* | glume base | c | 7 | 11 | 35 | 30 | **83** |
| *Triticum monococcum* | glume base | uc |  | 1 | 1 |  | **2** |
| *Triticum* sp. | grain | c | 1 |  | 4 |  | **5** |
| *Triticum* sp. | glume base | c | 2 | 1 | 47 | 9 | **59** |
| *Triticum* sp. | culm | uc |  | 2 |  |  | **2** |
| **Pulses** |  |  |  |  |  |  |  |
| *Pisum* sp. | seed | c | 1 |  |  |  | **1** |
| *Vicia* cf. *sativa* | seed | uc | 5 |  |  |  | **5** |
| **Flax** |  |  |  |  |  |  |  |
| *Linum usitatissimum* | seed | c | 17 |  |  | 4 | **21** |
| *Linum usitatissimum* | boll | uc |  |  |  | 2 | **2** |
| **Arable weeds (Echinochloa)** |  |  |  |  |  |  |  |
| *Echinochloa crus-galli* | grain | c | 126 | 30 | 43 | 68 | **267** |
| *Echinochloa crus-galli* | grain | uc |  |  |  | 24 | **24** |
| **Arable weeds, ruderal and nitrophilous plants** | |  |  |  |  |  |  |
| *Avena* sp. | grain | c |  | 1 | 1 |  | **2** |
| *Chenopodium album* | seed | c | 14 | 68 | 1 |  | **83** |
| *Chenopodium album* | seed | uc |  |  | 1 | 23 | **24** |
| *Chenopodium ficifolium* | seed | uc |  |  | 3 |  | **3** |
| *Daucus carota* | seed | uc |  |  | 1 | 35 | **36** |
| *Fallopia convolvulus* | seed | c |  | 6 |  | 2 | **8** |
| *Fallopia convolvulus* | seed | uc |  |  | 8 |  | **8** |
| *Galeopsis* cf. *tetrahit* | seed | uc |  | 1 |  |  | **1** |
| *Galium aparine* | seed | c |  |  |  | 5 | **5** |
| *Galium* sp. | seed | uc |  |  |  | 1 | **1** |
| *Hypericum perforatum* | seed | uc |  |  |  | 5 | **5** |
| *Persicaria maculosa* | seed | uc |  | 1 |  | 4 | **5** |
| *Portulaca oleracea* | seed | c | 1 |  |  |  | **1** |
| *Portulaca oleracea* | seed | uc |  | 1 |  | 3 | **4** |
| *Silene latifolia* | seed | uc |  |  | 2 |  | **2** |
| *Solanum nigrum* | seed | c | 1 |  |  |  | **1** |
| *Stachys annua* | seed | uc |  | 3 |  | 2 | **5** |
| *Verbena officinalis* | seed | uc |  |  |  | 6 | **6** |
| *Urtica dioica* | seed | uc |  |  | 6 |  | **6** |
| **Grassland plants** |  |  |  |  |  |  |  |
| *Agrimonia eupatoria* | seed | c |  | 1 |  |  | **1** |
| *Agrimonia eupatoria* | seed | uc |  |  | 2 | 1 | **3** |
| *Ajuga chamaepitys* | seed | c |  |  |  | 2 | **2** |
| *Crupina* cf. *vulgaris* | seed | uc |  | 1 | 3 |  | **4** |
| *Fragaria vesca* | seed | uc | 2 |  | 1 |  | **3** |
| *Medicago* cf. *polymorpha* | seed | uc |  |  | 1 | 2 | **3** |
| *Potentilla argentea* | seed | uc |  |  |  | 4 | **4** |
| *Potentilla* sp. | seed | uc |  |  | 7 | 1 | **8** |
| *Ranunculus acris* | seed | c |  |  |  | 3 | **3** |
| *Ranunculus acris* | seed | uc |  | 3 |  |  | **3** |
| *Valerianella dentata* | seed | uc |  |  |  | 4 | **4** |
| *Valerianella* sp. | seed | uc |  |  | 1 |  | **1** |
| **Wetland plants** |  |  |  |  |  |  |  |
| *Carex* cf. *acuta* | seed | uc |  |  |  | 24 | **24** |
| *Carex* cf. *acutiformis* | seed | uc |  |  |  | 16 | **16** |
| *Carex distans* | seed | c |  | 4 |  |  | **4** |
| *Carex distans* | seed | uc | 2 | 1 |  | 616 | **619** |
| *Carex* cf. *flava* | seed | c | 4 |  |  |  | **4** |
| *Carex* cf*. flava* | seed | uc | 1 |  |  |  | **1** |
| *Carex hirta* | seed | c | 2 |  |  |  | **2** |
| *Carex hirta* | seed | uc |  |  | 2 | 2 | **4** |
| *Carex* sp. | seed | c | 3 |  |  |  | **3** |
| *Carex* sp. | seed | uc | 2 | 3 | 26 | 41 | **72** |
| *Lycopus europaeus* | seed | uc |  |  |  | 9 | **9** |
| *Persicaria hydropiper* | seed | uc |  | 2 |  |  | **2** |
| *Persicaria lapathifolia* | seed | c |  |  |  | 1 | **1** |
| *Persicaria lapathifolia* | seed | uc |  |  |  | 1 | **1** |
| *Ranunculus repens* | seed | c | 2 |  |  |  | **2** |
| *Ranunculus sardous* | seed | c |  |  |  | 1 | **1** |
| *Ranunculus sardous* | seed | uc |  |  | 1 |  | **1** |
| *Scirpus* sp. | seed | uc |  |  |  | 1 | **1** |
| *Stachys* cf*. palustris* | seed | uc |  | 1 |  | 13 | **14** |
| **Trees and Shrubs (fruits, nuts and buds)** | |  |  |  |  |  |  |
| *Cornus mas* | seed | c |  |  |  | 2 | **2** |
| *Cornus mas* | seed | uc | 4 | 3 | 8 | 1 | **16** |
| *Corylus avellana* | shell | uc |  | 20 | 4 |  | **24** |
| *Pyrus sp.* | seed | c |  |  | 1 |  | **1** |
| *Pyrus/malus* | seed | uc |  | 1 |  |  | **1** |
| *Quercus* cf. *robur* | bud | uc |  | 1 | 3 |  | **4** |
| *Quercus* sp. | seed | c |  | 2 |  |  | **2** |
| *Quercus* sp. | acorn base | c | 4 |  |  |  | **4** |
| *Quercus* sp. | acorn base | uc | 1 |  |  | 11 | **12** |
| *Rubus* *fruticosus* | seed | uc | 1 | 15 | 13 | 7 | **36** |
| *Rubus* sp. | seed | uc |  | 1 | 2 | 6 | **9** |
| *Vitis vinifera* ssp*. sylvestris* | seed | uc |  | 10 | 5 | 4 | **19** |
| **Tot.** |  |  | **213** | **204** | **264** | **1028** | **1709** |
