# Supplementary material for: Multiproxy analysis of stabling layers in four middle bronze age byre-houses from the site of Oppeano 4D (Verona, Italy)
Source: PLoS One. 2025 May 22;20(5):e0323724. doi: 10.1371/journal.pone.0323724 (PMC12097577; doi:10.1371/journal.pone.0323724)
Supplement: SM 6 Biomarkers — Table 1. SM6 Acronyms, common and IUPAC names of the investigated steroids and the selected internal standard with the related m/z ions used for GC-MS analysis. Table 2. SM6 Steroids concentrations (ng/g) in the 24 samples from structures C, G and F of Oppeano site. COP = Coprostanol; EPI-COP = Epi-coprostanol; CHL = Cholesterol; CHN = 5α-cholestanol; CHONE = Cholestanone; 24-COP = 5β-stigmastanol; 24-EPI-COP = Epi-5β-stigmastanol; CAMP = Campesterol; STGR = Stigmasterol; β-SIT = β-sitosterol; STGN = 5α-stigmastanol. < MDL = lower than the method detection limit. Table 3. SM6 Steroids concentrations (µg/g) of crops and leaves of trees representative of the Bronze Age period. COP = Coprostanol; EPI-COP = Epi-coprostanol; CHL = Cholesterol; CHN = 5α-cholestanol; CHONE = Cholestanone; 24-COP = 5β-stigmastanol; 24-EPI-COP = Epi-5β-stigmastanol; CAMP = Campesterol; STGR = Stigmasterol; ERGO = Ergosterol; β-SIT = β-sitosterol; STGN = 5α-stigmastanol. (DOCX) [file pone.0323724.s006.docx]

**Supplementary Material 6** of the manuscript Nicosia et al.

Samples were analyzed as follows.

Soils and plant materials were previously freeze-dried to remove humidity and grounded. Amounts between 2-4 g of the dried sample were extracted through an accelerated solvent extractor (ASE350, Dionex Thermo Fisher Scientific, Waltham, MA, USA) equipped with 22 mL stainless steel cells. Cells were filled with sample, decontaminated diatomaceous earth as dispersant material and 400 ng of internal standard (Cholesterol-25,26,27-^13^C_3_). The extraction was conducted with dichlorometane (DCM, stabilised with amylene, assay > 99.9 %, Romil Ltd, Cambridge, UK) at 150 °C, 1500 psi, and with two 5 min. static cycles. The extracted liquid (~40 mL) was concentrated to ~1 mL under a gentle nitrogen flux. Fast purification was performed using Pasteur pipettes filled with glass wool and 4 cm of 100–200 mesh Florisil^®^ (Thermo Fisher Scientific, Waltham, MA, USA); 10 mL of DCM were used to previously decontaminate the Florisil^®^ and, then, samples were eluted using other 10 mL of dichlorometane. The purified extract (~10 mL) was finally evaporated it until dry, resumed with 100 µL of dichlorometane and transferred into GC vials with a 250 μL glass insert. Derivatization was carried out spiking 100 μL of N,O-Bis(trimethylsilyl)trifluoroacetamide (BSTFA) + 1% TMCS (Alfa Aesar, Haverhill, MA, USA) to the concentrated extract and the solution was heated at 70 °C for 1 hour.

GC-MS analysis was carried out using a 7890A GC system coupled with a 5975C MSD single quadrupole spectrometer (Agilent, Santa Clara, CA, USA) equipped with a HP-5MS capillarity column (60 m x 250 μm x 0.25 μm - Agilent). The GC oven was programmed as follows: starting point 150 °C, then 30 °C /min to 220 °C, then 0.7 °C /min to 275 °C, and then 10 °C /min to 300 °C. The He flow was set at 1.2 mL/min, the injection was performed in splitless mode, the temperature of the injector was set at 290 °C. The interface, electron impact (EI) source and quadrupole temperatures were set at 300 °C, 230 °C and 150°C, respectively. The EI source was set to 70 eV. The analysis was performed in Single Ion Monitoring (SIM) mode by checking two characteristic m/z ions for each investigated steroid, as reported in the table below, to check the presence of possible interferents in the analysis (see Battistel et al. 2015). Four procedural blanks were analysed to evaluate the method limit of detection (defined as 3 times the standard deviation), the limit of quantification (defined as 10 times the standard deviation), and to correct the concentration values obtained.

**Tab. 1 SM6** Acronyms, common and IUPAC names of the investigated steroids and the selected internal standard with the related m/z ions used for GC-MS analysis.

| Acronym | Common name | IUPAC name | GC-MS m/z ions |
| --- | --- | --- | --- |
| COP | Coprostanol | 5β-Cholestan-3β-ol | 215, 370 |
| EPI-COP | Epi-coprostanol | 5β-Cholestan-3α-ol | 215, 370 |
| CHL | Cholesterol | Cholest-5en-3β-ol | 329, 368 |
| CHN | 5α-cholestanol | 5α-Cholestan-3β-ol | 215, 445 |
| CHONE | Cholestanone | 5α-Cholesten-3-one | 316, 386 |
| 24-COP | 5β-stigmastanol | 24-Ethyl-5β-cholestan-3β-ol | 215, 398 |
| 24-EPI-COP | Epi-5β-stigmastanol | 24-Ethyl-5β-cholestan-3α-ol | 215, 398 |
| CAMP | Campesterol | 24-Methyl-cholest-5en-3β-ol | 343, 382 |
| STGR | Stigmasterol | 24-Ethyl-Cholesta-5,22 en dien-3β-ol | 484, 394 |
| β-SIT | β-sitosterol | 24-Ethyl-cholest-5en-3β-ol | 396, 357 |
| STGN | 5α-stigmastanol | 24-Ethyl-5α-cholestan-3β-ol | 215, 473 |
| CHL-13 (IS) | Cholesterol-25,26,27-^13^C_3_ | 3β-(25,26,27-^13^C_3_) Cholest-5-en-3-ol | 332, 371 |

**Tab. 2 SM6** Steroids concentrations (ng/g) in the 24 samples from structures C, G and F of Oppeano site. COP = Coprostanol; EPI-COP = Epi-coprostanol; CHL = Cholesterol; CHN = 5α-cholestanol; CHONE = Cholestanone; 24-COP = 5β-stigmastanol; 24-EPI-COP = Epi-5β-stigmastanol; CAMP = Campesterol; STGR = Stigmasterol; β-SIT = β-sitosterol; STGN = 5α-stigmastanol. < MDL = lower than the method detection limit.

| Samples | | Steroid concentration (ng/g) in Oppeano structures | | | | | | | | | | | |
| --- | --- | --- | --- | --- | --- | --- | --- | --- | --- | --- | --- | --- | --- |
| Structure | **N°** | COP | EPI-COP | CHL | CHN | CHONE | 24-COP | 24-EPI-COP | CAMP | STGR | β-SIT | STGN | Total steroids |
| G | OP_21 | 90 | 75 | 249 | 339 | 379 | 688 | 603 | 119 | 271 | 913 | 2047 | 5771 |
|  | OP_22 | 329 | 213 | 392 | 605 | 1017 | 2069 | 1684 | 98 | 352 | 1162 | 3418 | 11339 |
|  | OP_23 | 415 | 329 | 367 | 577 | 1350 | 2104 | 2906 | < MDL | 344 | 801 | 3292 | 12485 |
|  | OP_24 | 461 | 667 | 147 | 287 | 2906 | 2862 | 4487 | 22 | 202 | 545 | 1652 | 14239 |
|  | OP_25 | 89 | 62 | 516 | 622 | 162 | 864 | 884 | 141 | 452 | 1158 | 2672 | 7624 |
|  | OP_26 | 299 | 181 | 493 | 686 | 1746 | 2395 | 2482 | 118 | 421 | 930 | 2880 | 12631 |
|  | OP_27 | 210 | 122 | 443 | 585 | 635 | 1109 | 975 | 90 | 283 | 577 | 2683 | 7712 |
|  | OP_32 | 409 | 419 | 318 | 554 | 1262 | 3740 | 6817 | 81 | 428 | 836 | 3621 | 18484 |
|  | OP_28 | 1371 | 622 | 152 | 356 | 8872 | 5474 | 3022 | 66 | 46 | 404 | 2610 | 22996 |
|  | OP_29 | 195 | 173 | 111 | 178 | 803 | 771 | 1427 | 56 | 42 | 417 | 1409 | 5583 |
| C | OP_36 | 106 | 51 | < MDL | 4.73 | < MDL | 1107 | 1068 | < MDL | < MDL | 13 | 7 | 2357 |
|  | OP_37 | 1 | 0.77 | < MDL | < MDL | 0.22 | 15 | 24 | < MDL | < MDL | < MDL | 3 | 43 |
|  | OP_38 | 0.13 | 0.10 | < MDL | < MDL | < MDL | 3 | 5 | 0.20 | < MDL | < MDL | < MDL | 9 |
|  | OP_39 | 68 | 25 | < MDL | 1.20 | 10 | 1046 | 633 | 3 | 26 | 35 | 8 | 1855 |
|  | OP_40 | 93 | 79 | < MDL | 1.60 | 7 | 2398 | 3187 | < MDL | < MDL | 39 | 38 | 5843 |
|  | OP_41 | 0.81 | 0.75 | < MDL | < MDL | < MDL | 21 | 29 | 0.19 | < MDL | < MDL | 0.21 | 52 |
|  | OP_42 | 0.02 | 0.01 | < MDL | < MDL | < MDL | 0.25 | 0.20 | < MDL | < MDL | < MDL | < MDL | 0.48 |
|  | OP_43 | 13 | 19 | < MDL | 0.90 | < MDL | 150 | 350 | 0.75 | < MDL | 5 | 3 | 542 |
|  | OP_44 | 1 | 2 | 2 | < MDL | < MDL | 23 | 76 | 2 | < MDL | < MDL | < MDL | 106 |
| F | OP_48 | 5 | 3 | < MDL | 0.49 | < MDL | 12 | 13 | 0.71 | < MDL | < MDL | 4 | 38 |
|  | OP_49 | 6 | 5 | < MDL | < MDL | < MDL | 26 | 38 | 1 | < MDL | < MDL | 3 | 80 |
|  | OP_50 | 153 | 63 | 36 | 25 | 73 | 1080 | 655 | 15 | < MDL | 2957 | 446 | 5503 |

**Tab. 3 SM6** Steroids concentrations (µg/g) of crops and leaves of trees representative of the Bronze Age period. COP = Coprostanol; EPI-COP = Epi-coprostanol; CHL = Cholesterol; CHN = 5α-cholestanol; CHONE = Cholestanone; 24-COP = 5β-stigmastanol; 24-EPI-COP = Epi-5β-stigmastanol; CAMP = Campesterol; STGR = Stigmasterol; ERGO = Ergosterol; β-SIT = β-sitosterol; STGN = 5α-stigmastanol.

| Samples | | | Steroid concentration (µg/g) in plant materials | | | | | | | | | | | | |
| --- | --- | --- | --- | --- | --- | --- | --- | --- | --- | --- | --- | --- | --- | --- | --- |
| Abbreviation | Specie | **Portion** | COP | EPI-COP | CHL | CHN | CHONE | 24-COP | 24-EPI-COP | CAMP | STGR | ERGO | β-SIT | STGN | Total steroids |
| *Trit. Ae.* | *Triticum aestivum* | ear |  |  | 2 |  |  |  |  | 16 | 19 | 4.7 | 61 | 5.9 | 109 |
| *Trit. M.* | *Triticum monococcum* | stem |  |  | 2.5 |  |  |  |  | 33 | 15 | 8.3 | 125 | 4.4 | 189 |
|  |  | ear |  |  | 2 |  |  |  |  | 16 | 19 | 4.7 | 61 | 5.9 | 109 |
| *Panic. M.* | *Panicum miliaceum* | stem |  |  | 6.8 |  |  |  |  | 34 | 9 | 5.0 | 47 | 0.8 | 102 |
|  |  | leaf |  |  | 63 | 2 |  |  |  | 81 | 75 | 45 | 148 | 4.2 | 417 |
|  |  | inflorescence |  |  | 39 | 0.6 |  |  |  | 11 | 32 | 16 | 80 | 2.1 | 181 |
|  |  | seed |  |  | 7.7 |  |  |  |  | 6.7 | 7.4 | 3.1 | 37 |  | 62 |
| *Hord. V* | *Hordeum vulgare* | chaff |  |  | 2.2 |  |  |  |  | 13 | 24 | 4.4 | 60 | 2.6 | 106 |
| *Vic. S* | *Vicia sativa* | whole plant |  |  | 1.5 |  |  |  |  | 29 | 51 | 20 | 276 | 10 | 388 |
| *Ulmus* | *Ulmus* | leaf |  |  |  |  |  | 0.6 |  | 6 | 1.7 | 3.7 | 213 | 2.1 | 227 |
| *Ulmus M.* | *Ulmus minor* | leaf |  |  |  |  |  |  |  | 6.9 | 6.7 |  | 209 | 5.3 | 228 |
| *Cor. Av.* | *Corylus avellana* | leaf |  |  | 0.6 |  |  |  |  | 4 | 8.4 | 6.4 | 266 | 2.9 | 289 |
| *Quercus* | *Quercus* | leaf |  |  |  |  |  |  |  | 1.3 | 1.3 | 1.1 | 242 | 2.4 | 248 |
| *Fraxinus* | *Fraxinus* | leaf |  |  | 0.8 |  |  |  |  | 1 | 1 |  | 96 | 0.8 | 100 |
| *Salix* | *Salix* | leaf |  |  | 0.8 |  |  |  |  | 1.2 | 1.1 | 5.2 | 446 | 1.1 | 456 |
| *Carp.* | *Carpinus betulus* | leaf |  |  | 0.7 |  |  |  |  | 4.4 | 6.8 | 5.1 | 317 | 4.1 | 339 |
